# Modelling cadmium‐induced cardiotoxicity using human pluripotent stem cell‐derived cardiomyocytes

**DOI:** 10.1111/jcmm.13702

**Published:** 2018-07-11

**Authors:** Jiaxi Shen, Xiaochen Wang, Danni Zhou, Tongyu Li, Ling Tang, Tingyu Gong, Jun Su, Ping Liang

**Affiliations:** ^1^ Key Laboratory of combined Multi‐organ Transplantation Ministry of Public Health The First Affiliated Hospital Zhejiang University School of Medicine Hangzhou China; ^2^ Institute of Translational Medicine Zhejiang University Hangzhou China; ^3^ The First Affiliated Hospital Zhejiang University School of Medicine Hangzhou China

**Keywords:** apoptosis, cadmium‐induced cardiotoxicity, electrophysiology, hPSC‐CMs, MAPK, PI3K/Akt

## Abstract

Cadmium, a highly ubiquitous toxic heavy metal, has been widely recognized as an environmental and industrial pollutant, which confers serious threats to human health. The molecular mechanisms of the cadmium‐induced cardiotoxicity (CIC) have not been studied in human cardiomyocytes at the cellular level. Here we showed that human pluripotent stem cell‐derived cardiomyocytes (hPSC‐CMs) can recapitulate the CIC at the cellular level. The cadmium‐treated hPSC‐CMs exhibited cellular phenotype including reduced cell viability, increased apoptosis, cardiac sarcomeric disorganization, elevated reactive oxygen species, altered action potential profile and cardiac arrhythmias. RNA‐sequencing analysis revealed a differential transcriptome profile and activated MAPK signalling pathway in cadmium‐treated hPSC‐CMs, and suppression of P38 MAPK but not ERK MAPK or JNK MAPK rescued CIC phenotype. We further identified that suppression of PI3K/Akt signalling pathway is sufficient to reverse the CIC phenotype, which may play an important role in CIC. Taken together, our data indicate that hPSC‐CMs can serve as a suitable model for the exploration of molecular mechanisms underlying CIC and for the discovery of CIC cardioprotective drugs.

## 1 INTRODUCTION

Cadmium (Cd), a highly ubiquitous toxic heavy metal, has been widely recognized as an environmental and industrial pollutant, which confers serious threats to human health and thus it ranks 8th on the Agency for Toxic Substances and Disease Registry List of Hazardous Substances (ATSDR, 2005).^1^, ^2^ Cd exposure by humans has dramatically increased, which are mainly through workplace, cigarette smoke, contaminated food and water.^3^ When Cd ions are assimilated, they cannot be effectively removed through a biochemical mechanism, which accumulate in the human body over time with a half‐life time of 15‐30 years.^4^ Cd ions may enter cells through ion channels and transporters. As a nonessential element, there are no Cd‐specific ion channels or transported proteins on the cell membrane, and Ca^2+^ channel, Fe^2+^ channel or Zn^2+^ channel systems have been described that are capable of transporting Cd ions across the cell membrane.^5^, ^6^ When entering cells, free Cd may be bound to metal binding proteins such as metallothionein (MT) to form protein‐bound ions as the major detoxification system.

Cadmium can damage multiple organs in human body and the primary targets include kidney, lung, liver, testes, bones and cardiovascular system.^2^, ^7^, ^8^, ^9^, ^10^, ^11^, ^12^, ^13^, ^14^, ^15^, ^16^ Previous studies have suggested an association between Cd exposure and risk of cardiovascular diseases such as myocardial infarction, peripheral arterial disease, cardiomyopathy, hypertension and arteriosclerosis, stroke and heart failure.^17^, ^18^, ^19^, ^20^, ^21^, ^22^, ^23^ Cadmium‐induced cardiotoxicity (CIC) has been studied mainly in cultured cardiomyocytes isolated from murine models.^24^, ^25^, ^26^, ^27^, ^28^, ^29^, ^30^ However, animal models cannot accurately recapitulate the CIC because of the large difference in gene expression profile, electrophysiology and contractile features, and responses to specific therapies between murine and human cardiac systems. Human pluripotent stem cell‐derived cardiomyocytes (hPSC‐CMs) offers a human‐based, physiology‐relevant and scalable cell source for disease modelling and drug screening.^31^, ^32^, ^33^, ^34^, ^35^Several studies have demonstrated the reliability of hPSC‐CMs to study drug‐induced cardiotoxicity in a dish, which encourages us to investigate the CIC using hPSC‐CMs.^36^, ^37^, ^38^, ^39^, ^40^, ^41^, ^42^, ^43^, ^44^

In this study, we utilized hPSC‐CM as a unique platform to investigate the molecular mechanisms of CIC. We observed cellular phenotype in Cd‐treated hPSC‐CMs, including reduced cell viability, increased apoptosis, cardiac sarcomeric disorganization, elevated reactive oxygen species (ROS), altered electrophysiology and cardiac arrhythmias, when compared to control cells. RNA‐Sequencing (RNA‐Seq) analyses revealed a differential transcriptome profile and activated MAPK signalling pathway in Cd‐treated hPSC‐CMs, and suppression of P38 MAPK rescued CIC phenotype. We further identified that suppression of PI3K/Akt signalling pathway is sufficient to reverse the CIC phenotype, which may play an important role in CIC. Taken together, it is the first in vitro stem cell model to study CIC, which provides a suitable model for the exploration of molecular mechanisms underlying CIC and for the discovery of CIC cardioprotective drugs.

## 2 MATERIALS AND METHODS

### 2.1 Culture and maintenance of H9 human embryonic stem cells

H9 human embryonic stem cells (hESCs) were utilized in this study, which were obtained from WiCELL (Madison, WI). Cells were cultured in feeder‐free mTeSR1 (STEMCELL Technologies) media on matrigel‐coated (Corning) plates at 37°C with 5% (vol/vol) CO_2_. The media were daily changed, and cells were passaged every 3‐4 days using StemPro Accutase (Gibco).

### 2.2 Cardiac differentiation

H9 hESCs were differentiated into cardiomyocytes (CMs) using a 2D monolayer differentiation protocol as previously described.^45^ Briefly, ~10^5^ undifferentiated cells were dissociated and re‐plated into matrigel‐coated 6‐well plates. Cells were cultured and expanded to 85% cell confluence and then treated for 2 days with 6 μmol/L CHIR99021 (Axon Medchem) in RPMI and B‐27 supplement minus insulin (RPMI+B27‐Insulin) (Gibco) to activate Wnt signalling pathway. On day 2, cells were placed in RPMI+B27‐Insulin with CHIR99021 removal. On days 3‐4, cells were treated with 5 μmol/L IWR‐1 (Axon Medchem) to inhibit Wnt signalling pathway. On days 5‐6, cells were removed from IWR‐1 treatment and placed in RPMI+B27‐Insulin. From day 7 onwards, cells were placed and cultured in RPMI and B‐27 supplement with insulin (RPMI+B27 + Insulin) (Gibco) until beating was observed. Cells were glucose starved for 3 days with RPMI+B27 + Insulin for the hPSC‐CM purification.^46^ H9‐CMs of Day 30‐40 after cardiac differentiation were utilized for cadmium induction and downstream functional assays in this study.

### 2.3 FACS analysis of hPSC‐CMs

Monolayer CMs were dissociated into single cells using 0.25% Tripsin‐EDTA (Gibco) for 5 minutes at 37°C. Cells were pelleted and fixed with 4%PFA (Sangon Biotech) for 10 minutes on ice. Every step was washed with PBS (Sangon Biotech) before sample centrifugation. Cells were stained with TNNT2 (Abcam) at 4°C, and FITC‐conjugated goat anti‐mouse IgG antibody (Invitrogen) was used as secondary antibody.

### 2.4 Immunofluorescent staining

Cells were fixed with 4% paraformaldehyde (PFA) (Sangon Biotech) for 15 minutes, permeabilized with 0.1% Triton X (Sangon Biotech) for 5 minutes, and blocked with 3% BSA (Sigma‐Aldrich) for 1 hour. Cells were subsequently stained with appropriate primary antibodies and AlexaFluor conjugated secondary antibodies (Life Technologies). Nuclei were stained with DAPI (Roche Diagnostics). For the staining of pluripotency markers, the primary antibodies were OCT4 (Santa Cruz Biotechnology), NANOG (Santa Cruz Biotechnology), SSEA‐4 (Abcam) and SOX2 (Abcam). For the staining of cardiac‐specific markers, the primary antibodies were TNNT2 (Abcam) and α‐actinin (Abcam). Pictures were taken with 63× objective on confocal microscope (Nikon, A1) using NIS‐Elements AR software (Nikon).

### 2.5 Alkaline phosphatase staining

Alkaline phosphatase (AP) staining was performed using the VECTOR Blue Alkaline Phosphatase Substrate Kit (Vector Laboratories) following the manufacturer's instructions.

### 2.6 Cell viability assay

H9‐CMs were cultured in 96‐well plate. Cell viability analyses were performed using CCK8 based in vitro cell proliferation and cytotoxicity assay kit (Beyotime) according to the manufacturer′s instructions. Cells were incubated in the presence of 10 μL CCK8 reagent per well for 3 hours. Absorbance at 450 nm was measured using an iMark™ microplate reader (Bio‐Rad).

### 2.7 TUNEL assay

Apoptosis of hESC‐CMs was measured using an In‐Situ Cell Death Detection Kit (Roche Diagnostics) in accordance with the manufacturer's instructions. Cells were co‐stained with TNNT2 (Abcam) as described above. Images were collected and analysed using an inverted microscope (Nikon, Eclipse Ti‐S).

### 2.8 Caspase‐3 activity assay

Caspase 3 activities were performed using caspase 3 Assay Kit (Beyotime) according to the manufacturer′s instructions. H9‐CMs were digested with Trypsin‐EDTA (Gibco) and collected by centrifugation at 600 *g* for 5 minutes at 4°C. The cell pellets were washed with DPBS (Gibco) and re‐suspended in 1 × lysis buffer at a concentration of 100 μL per 2 million cells, incubated on ice for 15 minutes and then centrifuged at 16 000‐20 000 g for 10‐15 minutes at 4°C. Appropriate amount of protein was put in a 96‐well plate, and 10 μL of Ac‐DEVD‐pNA (acetyl‐Asp‐Glu‐Val‐Asp p‐nitroanilide) (2 mmol/L) was added per well and then incubated for 60‐120 minutes at 37°C. Absorbance at 405 nm was read using a MD M5 SpectraMax reader (Molecular Devices).

### 2.9 High‐content imaging

H9‐CMs were cultured in Matrigel‐coated 24‐well plate. Time‐lapse live cell imaging was performed using an Operetta High‐Content Imaging System (PerkinElmer) at 20× magnification. Images were then analysed with Harmony4.1 (PerkinElmer).

### 2.10 Transmission electron microscopy

H9‐CMs were dissociated with Tripsin‐EDTA, scrapped into a 1.5‐mL microcentrifuge tube and centrifuged and then fixed with cold 2.5%‐glutaraldehyde in 0.1 mol/L phosphate buffer overnight at 4°C. The specimen was postfixed with 1% OsO_4_ in phosphate buffer and dehydrated by a graded series of ethyl‐alcohol (30%, 50%, 70%, 80%, 90%, 95% and 100%) for 15‐20 minutes at each step and then transferred to absolute acetone for 20 minutes. The specimen was placed in 1:1 mixture of absolute acetone and final spur resin mixture for 1 hour at room temperature, and transferred to 1:3 mixture of absolute acetone and final spur resin mixture for 3 hours, and then transferred to final spur resin mixture overnight. The specimen was placed in 1.5‐mL tube contained spur resin, heated at 70°C for more than 9 hours and sectioned using a LEICA EM UC7 ultratome. The sections were then stained with uranyl acetate and alkaline lead citrate for 5‐10 minutes. Pictures were observed using a transmission electron microscopy (Hitachi, Model H‐7650).

### 2.11 Reactive oxygen species (ROS) assay

Cellular levels of ROS in H9‐CMs were determined using a Reactive Oxygen Species Assay Kit (Beyotime) according to the manufacturer's instructions.

### 2.12 Electrophysiology

H9‐CMs were mechanically and enzymatically dissociated to obtain single cells, which were seeded on Matrigel‐coated glass coverslips (Warner Instruments). Cells with spontaneous beatings were selected, and action potentials were recorded using an EPC‐10 patch clamp amplifier (HEKA). Continuous extracellular solution perfusion was achieved using a rapid solution exchanger (Bio‐logic Science Instruments). Data were acquired using PatchMaster software (HEKA) and digitized at 1 kHz. Data analyses were performed using Igor Pro (Wavemetrics) and Prism (Graphpad). A TC‐344B heating system (Warner Instruments) was used to maintain the temperature at 35.5‐37°C. Tyrodes solution was used as the external solution containing 140 mmol/L NaCl, 5.4 mmol/L KCl, 1 mmol/L MgCl_2_, 10 mmol/L glucose, 1.8 mmol/L CaCl_2_ and 10 mmol/L HEPES (pH 7.4 with NaOH at 25°C). The internal solution contained 120 mmol/L KCl, 1 mmol/L MgCl_2_, 10 mmol/L HEPES, 3 mmol/L Mg‐ATP, and 10 mmol/L EGTA (pH 7.2 with KOH at 25°C).

Sodium and calcium currents were recorded from single H9‐CMs using the ruptured patch clamp technique with conventional voltage clamp protocols. For sodium current recordings, pipette solutions contained: 10 mmol/L NaCl, 135 mmol/L CsCl, 2 mmol/L CaCl_2_, 5 mmol/L Mg‐ATP, 5 mmol/L EGTA, and 10 mmol/L HEPES (pH 7.2 with CsOH). Bath solution contained: 50 mmol/L NaCl, 110 mmol/L CsCl, 1.8 mmol/L CaCl_2_, 1 mmol/L MgCl_2_, 10 mmol/L glucose, 10 mmol/L HEPES and 0.001 mmol/L Nifedipine (pH 7.4 with CsOH). For calcium current recordings, pipette solutions contained: 145 mmol/L CsCl, 5 mmol/L NaCl, 1 mmol/L CaCl_2_, 5 mmol/L Mg‐ATP, 5 mmol/L EGTA, and 10 mmol/L HEPES (pH 7.2 with CsOH). Bath solution contained: 160 mmol/L TEA‐Cl, 5 mmol/L CaCl_2_, 1 mmol/L MgCl_2_, 10 mmol/L glucose, 10 mmol/L HEPES, 0.01 mmol/L TTX, 2 mmol/L 4‐AP (pH 7.4 with CsOH). All currents were normalized to cell capacitance to obtain current density. Steady‐state activation and inactivation curves were fitted using a Boltzmann equation: *I*/*I*_max_ = A/{1.0 + exp [(*V*_1/2_ − *V*)/*k*]}, in which *V*_1/2_ is half‐maximum (in)activation potential and *k* is slope factor.

### 2.13 RNA‐sequencing

RNA purity was checked using the Nano Photometer® spectrophotometer (IMPLEN), and RNA concentration was measured using Qubit® RNA Assay Kit in Qubit® 2.0 Flurometer (Life Technologies). RNA integrity was assessed using the RNA Nano 6000 Assay Kit of the Bioanalyzer 2100 system (Agilent Technologies). The transcriptome library for sequencing was generated using VAHTSTMmRNA‐seq v2 Library Prep Kit for Illumina® (Vazyme Biotech) following the manufacturer's recommendations. The clustering of the index‐coded samples was used VAHTS RNA Adapters set1/set2 for Illumina® (Vazyme Biotech) according to the manufacturer's instructions. After clustering, the libraries were sequenced on Illumina HiseqXTen platform using (2 × 150 bp) paired‐end module. The raw images were transformed into raw reads by base calling using CASAVA (http://www.illumina.com/support/documentation.ilmn). Then, raw reads in a fastq format were first processed using in‐house perl scripts. Clean reads were obtained by removing reads with adapters, reads in which unknown bases were more than 5% and low quality reads (the percentage of low quality bases was over 50% in a read, we defined the low quality base to be the base whose sequencing quality was no more than 10). At the same time, Q20, Q30, GC content of the clean data were calculated. After initial quality control, the clean reads were mapped to the reference sequence by using TopHat2 software (v2.1.1). The alignment files generated by TopHat2 were input to the Cufflinks software (v2.2.1), which is a program for the comparative assembly of transcripts and the estimation of their abundance in a transcriptome sequencing experiment using the measurement unit FPKM (fragments per kilobase of transcript per million mapped reads). After using Cuffmerge program to merge transcripts of each sample in different materials and stages into a single gtf file that was used to identify differentially expressed genes, we used Cuffdiff program to find DEGs (differentially expressed genes). The differentially expressed genes were identified with *q* value ≤0.05 and a fold change of ≥2 between control and CdCl_2_‐treated H9‐CMs. Furthermore, cluster analysis, gene ontology (GO) enrichment analysis (GO::TermFinder), pathway enrichment analysis (KOBAS) and protein interaction analysis (based on StringDBdatabase) of differentially expressed genes were implemented if necessary.

### 2.14 Quantitative real‐time PCR (qPCR)

Total RNA isolation from hESC‐CMs was performed using RNeasy Mini Kit (Qiagen). RNA concentration was measured using UV spectrophotometry at 260 nm (Nanodrop 2000, Thermo Scientific). cDNA was obtained using the High Capacity cDNA Reverse transcription Kit (Applied Biosystems). qPCR was performed using SYBR Green PCR Master Mix (Takara). Primer sequences used in this study are listed in Table S1. Each reaction was run in triplicates using an Applied Biosystems Viia7 Dx (Thermo Fisher Scientific). Gene expression values were normalized to the average expression of housekeeping gene GAPDH.

### 2.15 Western blot

H9‐CMs were grown in 6‐well plates to 80% confluence, detached with TrypLE (Gibco) and then pelleted at 12 000 rpm for 3‐5 minutes at 4°C. After washing with DPBS (Sangon Biotech), the pellets were re‐suspended in 50‐100 μL lysis buffer. Lysates were placed on ice for 30 minutes, and then, the supernatants were collected after centrifuging at 12 000 rpm for 5 minutes. Protein concentration was measured using a BCA kit (Pierce). Western blot was performed using standard protocol with the following antibodies: caspase 3 (Cell Signaling Technology), phosphorylated P38 (Cell Signaling Technology), total P38 (Cell Signaling Technology), total c‐Myc (Cell Signaling Technology), phosphorylated Akt (Cell Signaling Technology) and total Akt (Cell Signaling Technology).

### 2.16 Compounds and solutions

All the chemicals used in the electrophysiological experiments were purchased from Sigma‐Aldrich. Cadmium chloride (CdCl_2_) was purchased from Sigma‐Aldrich, and stock solutions were prepared in 100 mmol/L in H_2_O. When CdCl_2_ induction performed, a new vial of stock solution was used and dilutions were prepared within 30 minutes of induction. Escalating doses of CdCl_2_ (0.1, 1, 3, 10, 30 and 100 μmol/L) were firstly used for the dose–response experiments. For downstream investigations in this study, H9‐CMs were all treated with 30 μmol/L CdCl_2_ for 24 hours, and functional assays were performed at this condition. PD0325901, SB203580, SP600125 and Ly294002 were all purchased from Selleck. Z‐VAD‐FMK, catechin hydrate and geldanamycin were all purchased from Beyotime. Detailed information of each compound used in this study is listed in Table S2.

### 2.17 Statistical analysis

Statistical significance was determined by unpaired 2‐tailed Student's *t*‐test to compare 2 groups and by one‐way ANOVA to compare multiple groups. A *P* value of < .05 was considered statistical significant. Data were shown as mean ± SEM and analysed by GraphPad Prism 6 (GraphPad Software).

## 3 RESULTS

### 3.1 Generation of H9 human embryonic stem cell‐derived cardiomyocytes (H9‐CMs)

H9 embryonic stem cells (H9) were utilized for generation of cardiomyocytes (CMs), which exhibited typical stem cell morphology and stained positive for pluripotency markers (Nanog, SSEA‐4, Oct4 and Sox2) and alkaline phosphatase (Figure S1A‐C). We used a 2D in vitro monolayer protocol to differentiate H9 hESCs into CMs, which can give rise to a yield of >90% after purification indicated by FACS analysis (Figure S1D,E, Videos [Link], [Link]). The monolayer H9 hESC‐derived cardiomyocytes (H9‐CMs) were then mechanically and enzymatically dissociated into single cells, which exhibited positive staining of cardiac markers of TNNT2 and α‐actinin (Figure S1F).

### 3.2 Cadmium‐induced morphological changes and apoptosis in H9‐CMs

**Figure 1:**
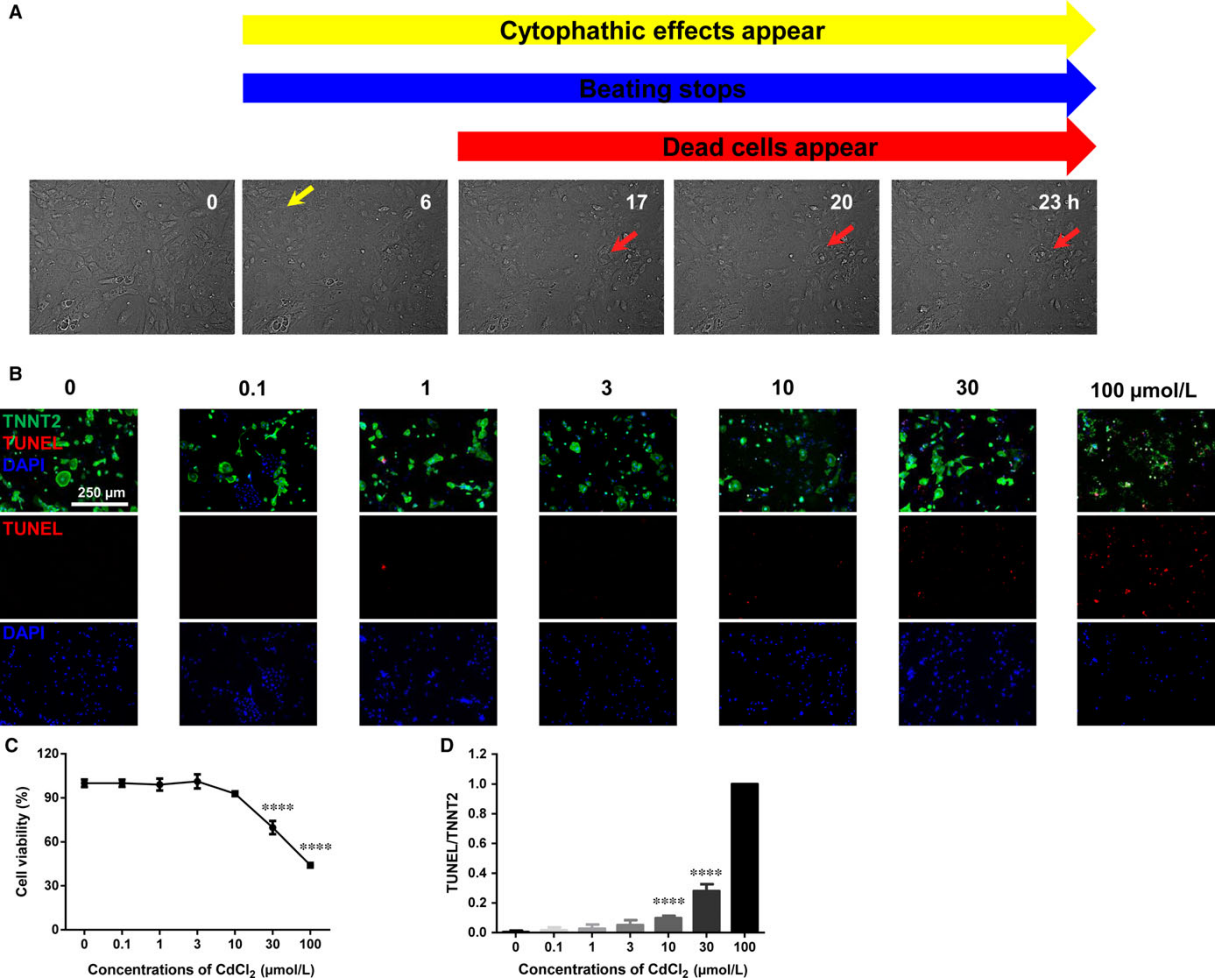
Cadmium‐induced morphological changes and apoptosis in H9‐CMs. A, Representative live cell imaging of H9‐CMs at 0, 6, 17, 20 and 23 h postinduction of 30 μmol/L CdCl_2_. Yellow arrow indicates cells became flat and three‐dimensional structure disappeared starting at 6 h postinduction. Red arrow indicates dead cells appeared starting at 17 h postinduction. B, Upper panel, Representative confocal images of co‐staining of TNNT2/TUNEL/DAPI in control and CdCl_2_‐treated H9‐CMs at different doses. Lower Panel, Representative confocal images of TUNEL staining in control and CdCl_2_‐treated H9‐CMs at different doses. Scale bar, 250 μm. C, Bar graph to compare the cell viability between control and CdCl_2_‐treated cells at different doses. ****P < .0001. D, Bar graph to compare the ratio of TUNEL/TNNT2 between control and CdCl_2_‐treated cells at different doses. ****P < .0001

We first sought to determine whether cadmium can induce morphological changes and cell death in H9‐CMs. Previous studies have investigated cadmium‐induced cytotoxicity in different cell types within a range of 0‐300 μmol/L.^47^, ^48^, ^49^, ^50^, ^51^, ^52^, ^53^ Therefore, cells were exposed to escalating concentrations of cadmium chloride (CdCl_2_) from 0.1 to 100 μmol/L for 24 hours, and significantly morphological changes appeared in cells treated with 30 and 100 μmol/L CdCl_2_ (Videos [Link], [Link], [Link]). To confirm this observation, the time‐lapse live cell imaging was performed using a High‐Content Imaging System in H9‐CMs treated with vehicle or 30 μmol/L CdCl_2_ for 24 hours. Starting at 6 hours postinduction, cytopathic effect was apparent with gradually cessation of beating, the cells became flat and 3‐dimensional structure disappeared (Figures 1A, S2 and S3). Dead cells were apparently observed starting at 17 hours postinduction (Figures 1A, S2 and S3). Moreover, we observed significantly reduced cell viability in CdCl_2_‐treated H9‐CMs in a dose‐dependent manner, when compared to control cells (Figure 1C). We next performed TUNEL assay to test whether CdCl_2_‐induced cell death relates to apoptosis. The cells were co‐stained with TNNT2, and we counted TUNEL‐positive cells in TNNT2‐positive cells. We found a significantly increased ratio of TUNEL‐positive cells in CdCl_2_‐treated cells in a dose‐dependent manner (Figure 1B,D). We selected 30 μmol/L as the induction concentration and H9‐CMs were all treated with 30 μmol/L CdCl_2_ treatment for 24 hours for downstream investigations in this study. In line with the TUNEL data, we observed a greatly higher protein expression of caspase 3 as well as a significantly increased caspase 3 activity in 30 μmol/L CdCl_2_‐treated cells (Figure S4A‐C). However, addition of Z‐VAD‐FMK, a specific‐caspase inhibitor, effectively rescued CdCl_2_‐induced phenotype including increased caspase 3 expression and activity, increased TUNEL signal and reduced cell viability (Figure S4A‐F). Taken together, these data suggest that H9‐CMs are susceptible to CdCl_2_ induction, resulting in dramatically morphological changes and increased cell apoptosis.

### 3.3 Cadmium‐induced elevated ROS in H9‐CMs

We next investigated whether ROS plays an important role in cadmium‐induced cell apoptosis in H9‐CMs. Consistent with previous studies,^24^, ^26^, ^27^, ^28^, ^29^ we observed a significantly higher level of ROS in 30 μmol/L CdCl_2_‐treated cells than controls, whereas catechin hydrate (CH), an anti‐oxidant, significantly reversed this phenotype (Figure S5A). Moreover, CdCl_2_‐induced cell loss and apoptosis were significantly rescued by addition of CH (Figure S5B,D). Taken together, these results suggested that elevated cellular ROS was associated with cadmium‐induced cell apoptosis and may be a key mediator of CIC.

### 3.4 Cadmium‐induced cardiac sarcomeric disorganization and ultrastructural changes in H9‐CMs

**Figure 2:**
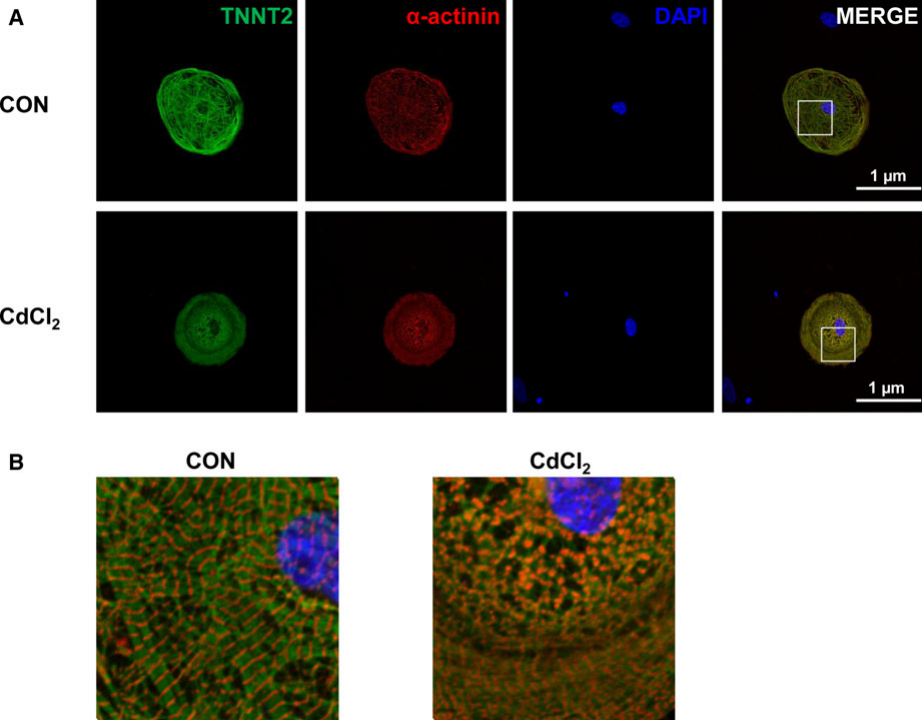
Cadmium‐induced cardiac sarcomeric disorganization and ultrastructural changes in H9‐CMs. A, Immunofluorescent staining of control and 30 μmol/L CdCl_2_‐treated H9‐CMs using cardiac‐specific markers TNNT2 (Green) and α‐actinin (Red). DAPI indicates nuclear staining (Blue). Scale bar, 1 μm. B, Enlarged view showing cardiac sarcomeres in control and CdCl_2_‐treated H9‐CMs

Previous studies have shown histological abnormalities of heart tissue in rat when treated with cadmium.^24^, ^25^, ^26^, ^27^, ^28^, ^29^, ^30^ We therefore assessed the extent of sarcomeric organization in H9‐CMs by immunostaining with TNNT2 and α‐actinin. As expected, control cells exhibited regular sarcomeric organization (Figures 2A,B and S6). By contrast, 30 μmol/L CdCl_2_‐treated cells showed a severe sarcomeric disorganization and disruption (Figures 2A,B and S6). To further assess the cell organelles in detail, we performed transmission electron microscope (TEM) in H9‐CMs. We observed apparent cardiac sarcomeres in control cells, which were absent in 30 μmol/L CdCl_2_‐treated ones. Moreover, CdCl_2_‐treated cells exhibited apoptotic signs including enlarged nucleocytoplasmic ratio and nuclear membrane shrinkage (Figure S7). Collectively, these data suggest that cadmium results in cardiac sarcomere disorganization and disruption as well as ultrastructural changes in H9‐CMs.

### 3.5 Cadmium‐induced action potential phenotype in H9‐CMs

**Figure 3:**
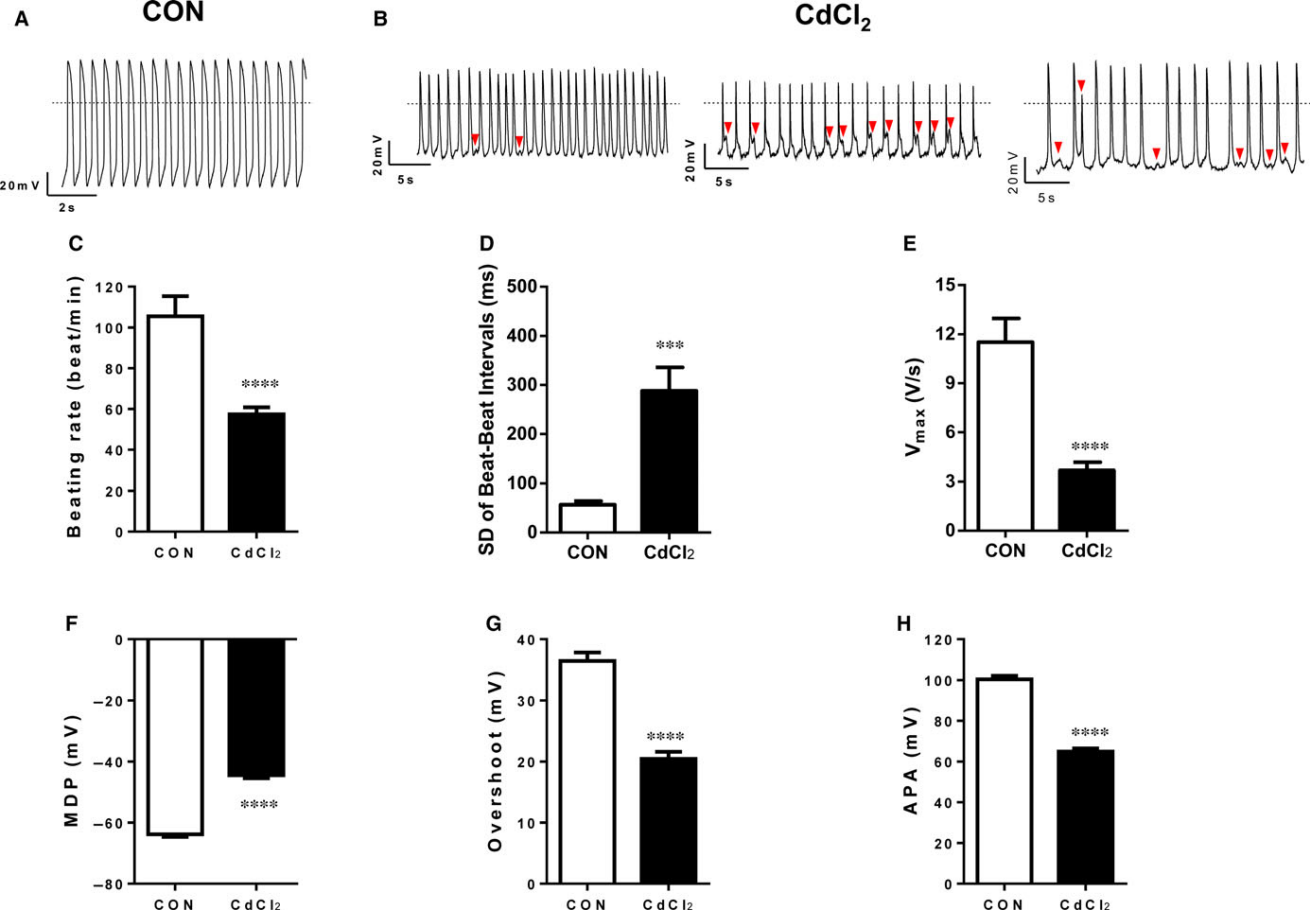
Cadmium‐induced electrophysiological phenotype in H9‐CMs. A and B, Representative action potential tracings of control and 30 μmol/L CdCl_2_‐treated H9‐CMs, respectively. Red arrows indicate arrhythmic events with EADs and DADs in CdCl_2_‐treated H9‐CMs. C‐H, Bar graph to compare beating rate, SD of beat–beat intervals, *V*_max_, MDP, overshoot and APA between control and CdCl_2_‐treated H9‐CMs, respectively. ***P < .001 and ****P < .0001

Cadmium exposure is associated with frontal T‐wave axis deviation, an easily detected subclinical marker of ventricular arrhythmias in individuals without heart disease.^54^ However, the effects of cadmium on in vitro cardiac electrophysiology have not been much studied yet. To investigate whether cadmium affects the electrophysiology of H9‐CMs, we performed patch clamp to record action potentials in these cells (Figure 3A,B and Table S3). Strikingly, an increased subfraction of 30 μmol/L CdCl_2_‐treated cells were observed to exhibit arrhythmias including early afterdepolarizations (EADs) and delayed afterdepolarizations (DADs), when compared to control ones (Figure 3A,B). The CdCl_2_‐treated cells also showed a greatly slower beating rate (Control: 105.50 ± 9.90; CdCl_2_: 57.40 ± 3.40), as well as an increased beat‐beat interval variability (Control: 55.50 ± 15.13 ms; CdCl_2_: 269.09 ±61.72 ms) in CdCl_2_‐treated cells, indicating an irregular heartbeat phenotype (Figure 3C,D). Interestingly, we observed a significantly reduced *V*_max_ in CdCl_2_‐treated cells (Control: 7.58 ± 1.35 V/s; CdCl_2_: 2.01 ± 0.25 V/s), reflecting a slower depolarization (Figure 3E). Moreover, CdCl_2_‐treated cells showed a significantly depolarized maximal diastolic potential (MDP) (Control: −64.5 ± 1.26 mV; CdCl_2_: −39.23 ± 1.21 mV), a significantly decreased overshoot (Control: 35.02 ± 3.27 mV; CdCl_2_: 20.09 ± 2.17 mV) and a significantly decreased action potential amplitude (APA) (Control: 99.49 ± 3.56 mV; CdCl_2_: 59.49 ± 2.07 mV) (Figure 3F‐H). Taken together, these data suggest that cadmium leads to action potential abnormalities and cardiac arrhythmias in H9‐CMs.

### 3.6 Cadmium‐induced ion channel remodelling in H9‐CMs

**Figure 4:**
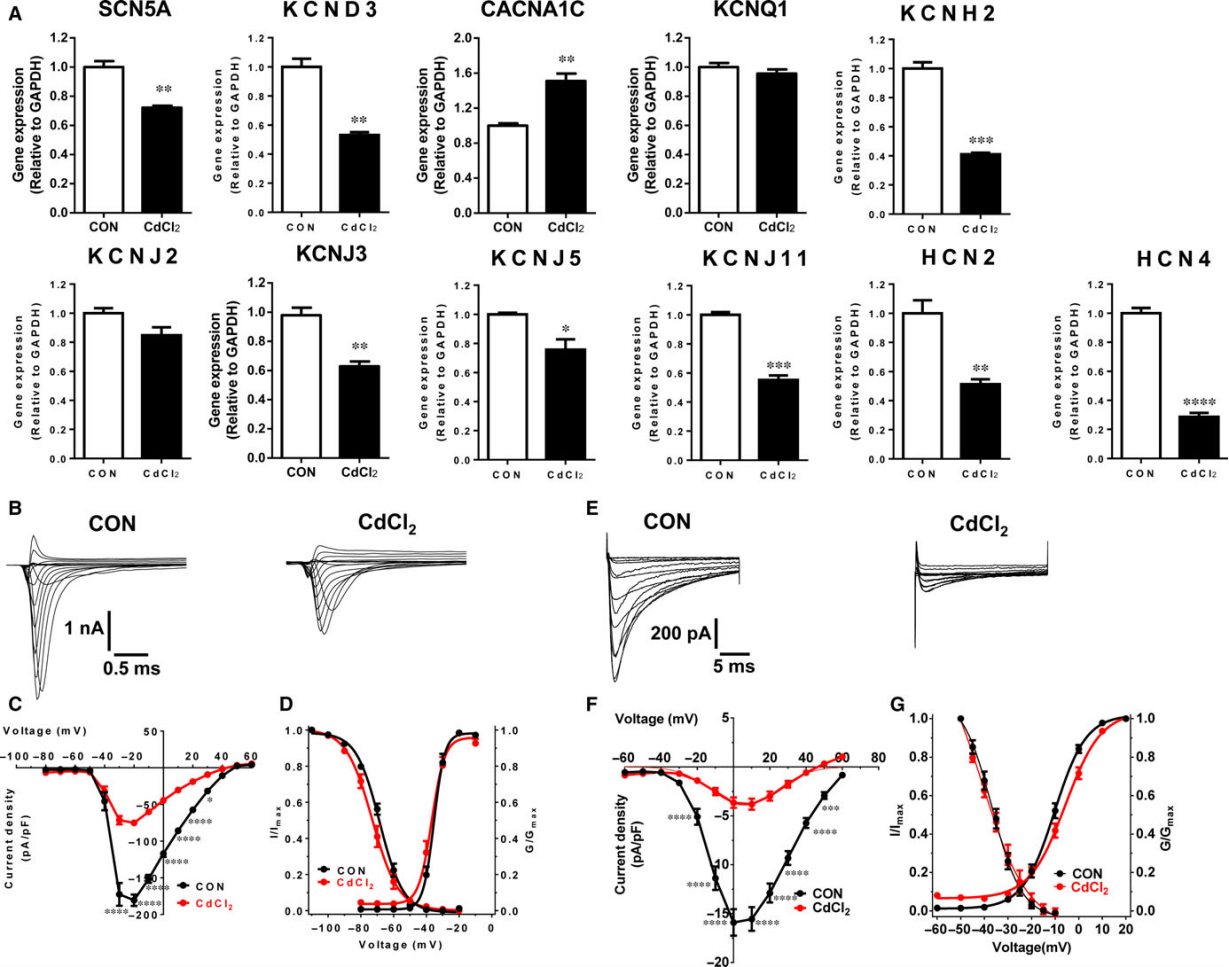
Cadmium‐induced ion channel remodelling in H9‐CMs. A, Bar graph to compare gene expression of major ion channels between control and 30 μmol/L CdCl_2_‐treated H9‐CMs, including SCN5A, KCND3, CACNA1C, KCNQ1, KCNH2, KCNJ2, KCNJ3, KCNJ5, KCNJ11, HCN2 and HCN4. *P < .05, **P < .01, ***P < .001 and ****P < .0001. B, Representative sodium current tracings isolated from control and 30 μmol/L CdCl_2_‐treated H9‐CMs. C, Comparison of sodium current–voltage relationship curve (IV curve) between control and CdCl_2_‐treated H9‐CMs. *P < .05 and ****P < .0001. D, Comparison of steady‐state activation and steady‐state inactivation of sodium current between control and CdCl_2_‐treated H9‐CMs. E, Representative calcium current tracings isolated from control and 30 μmol/L CdCl_2_‐treated H9‐CMs. F, Comparison of calcium current‐voltage relationship curve (IV curve) between control and CdCl_2_‐treated H9‐CMs. ***P < .001 and ****P < .0001. G, Comparison of steady‐state activation and steady‐state inactivation of calcium current between control and CdCl_2_‐treated H9‐CMs

We next performed qPCR to compare gene expression of a panel of cardiac ion channels between control and CdCl_2_‐treated H9‐CMs. The majority of the ion channel genes were decreasingly expressed in 30 μmol/L CdCl_2_‐treated cells, including SCN5A, KCND3, KCNH2, KCNJ3, KCNJ5, KCNJ11, HCN2 and HCN4 (Figure 4A and Table S1). By contrast, expression of CACNA1C was significantly increased (Figure 4A and Table S1). To investigate whether differential gene expression leads to functional consequences, we therefore isolated sodium (Na^+^) and calcium (Ca^2+^) currents from H9‐CMs by patch clamp recordings (Figures 4B,E and S8). We observed significantly reduced Na^+^ current density with unaltered steady‐state activation and inactivation properties in 30 μmol/L CdCl_2_‐treated cells, which is consistent with reduced expression of SCN5A and slower depolarization indicated by the reduced *V*_max_ in Figure 3E (Figure 4B‐D and Table S4). In line with a previous study,^55^ the Ca^2+^ current density was significantly decreased in 30 μmol/L CdCl_2_‐treated cells with comparable steady‐state activation and inactivation, although expression of CACNA1C was increased (Figure 4E‐G and Table S5). These results suggest that cadmium leads to ion channel remodelling at the cellular level in H9‐CMs.

### 3.7 RNA‐Seq analysis revealed differential transcriptome profile in cadmium‐treated H9‐CMs

**Figure 5:**
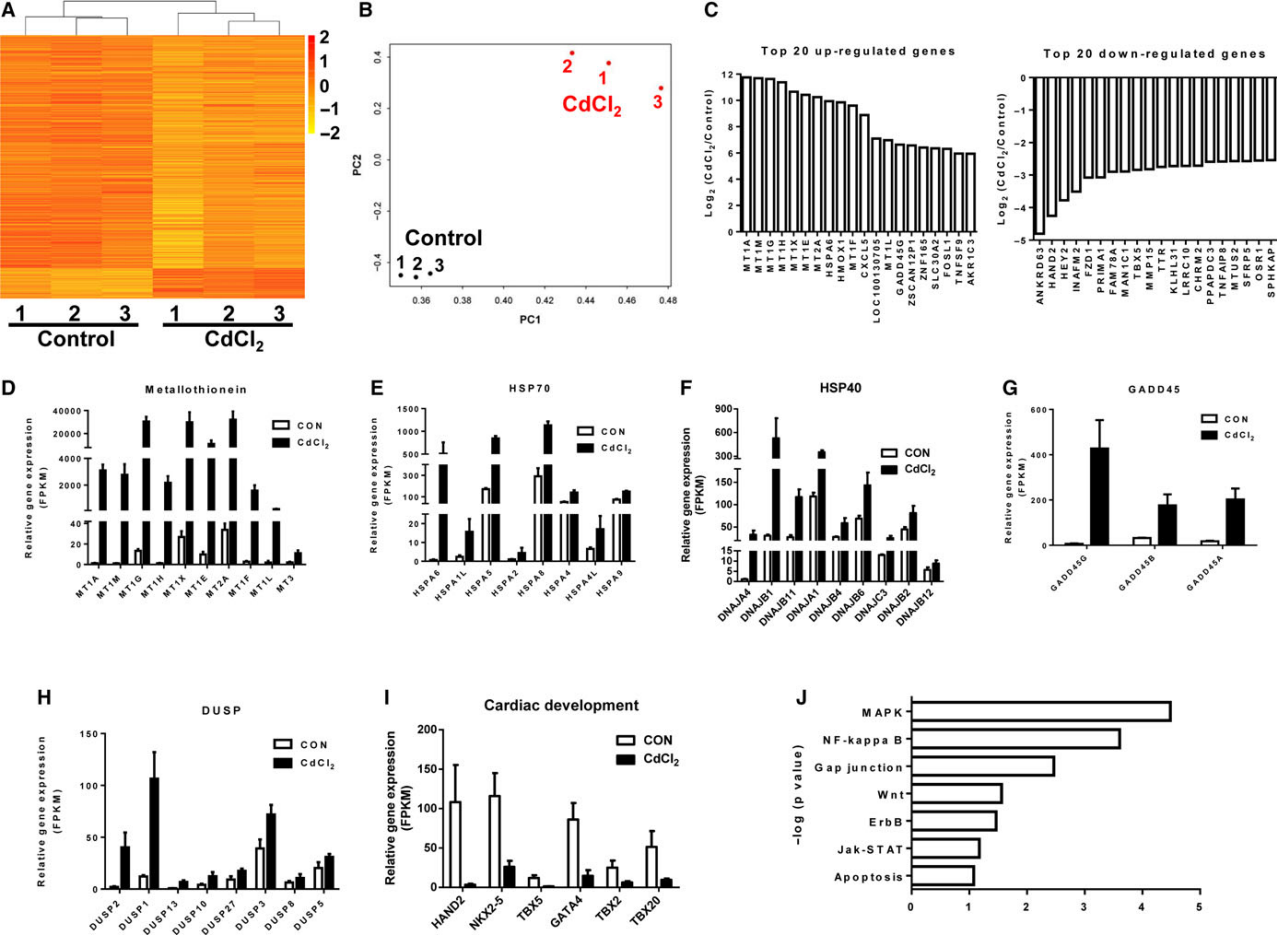
RNA‐Seq analysis revealed differential transcriptome profile in cadmium‐treated H9‐CMs. A, Heatmap demonstrating the differential gene expression pattern between control and 30 μmol/L CdCl_2_‐treated H9‐CMs. B, Principal component analysis (PCA) revealed that CdCl_2_‐treated samples clustered separately from control ones. C, Top 20 up‐ and down‐regulated genes showing the greatest differences in expression between CdCl_2_‐treated and control H9‐CMs. D‐I, Bar graphs to compare the FPKM values of metallothionein (MT), HSP70, HSP40, GADD45, DUSP, cardiac development genes between control and CdCl_2_‐treated H9‐CMs. J, Ingenuity pathway analysis (IPA) showing significantly altered signalling pathways in CdCl_2_‐treated H9‐CMs compared to control cells

To further analyse the molecular basis of CIC, we next performed genome‐wide RNA‐sequencing in both control and CdCl_2_‐treated H9‐CMs (Figure 5A). Principal component analysis (PCA) revealed that 30 μM CdCl_2_‐treated samples clustered together separately from control ones (Figure 5B). We found that 1978 genes of 22052 total genes (1361 up‐regulated, and 617 down‐regulated) were differentially expressed in CdCl_2_‐treated H9‐CMs (Figures 5C and S9). Several studies have implicated that increased expression of metallothionein (MT) and heat shock protein (HSP) genes was associated with cadmium‐induced cytotoxicity.^23^, ^24^, ^26^, ^56^ In line with the previous findings, we observed that the expression of numerous MT gene isoforms, heat shock protein family A (HSP70), DnaJ heat shock protein family (HSP40) was dramatically up‐regulated in CdCl_2_‐treated H9‐CMs, as compared to control cells (Figure 5D‐F). Growth arrest and DNA‐damage‐inducible protein (GADD45) genes, including GADD45G, GADD45B and GADD45A, were also increasingly expressed, which have been implicated as key mediators of apoptotic cardiomyocyte death (Figure 5G).^57^ Moreover, a cluster of dual specificity phosphatase 2 (DUSP) genes were significantly up‐regulated, including DUSP2, DUSP1, DUSP13, DUSP10, DUSP27, DUSP3, DUSP8 and DUSP5, which was associated with MAPK signalling pathway regulation after stress or mitogen stimulation in cardiomyocytes (Figure 5H).^58‐60^ However, the expression of genes encoding cardiac development and morphogenesis related transcription factors was significantly down‐regulated in the CdCl_2_‐treated H9‐CMs, as compared to the control cells, including HAND2, NK2 homeobox 5 (NKX2‐5), T‐box 5 (TBX5), GATA4, T‐box 2 (TBX2), T‐box 20 (TBX20) and HEY2 (Figure 5I). Interestingly, gene ontology (GO) analysis revealed that genes were positively enriched in “regulation of cell death,” “regulation of apoptotic process,” “regulation of programmed cell death,” “regulation of response to stimulus” and “regulation of response to stress” which are highly consistent with observed CIC (Figure S10 and Table S6). Notably, ingenuity pathway analysis (IPA) revealed significant up‐regulation of protein processing in mitogen‐activated protein kinase (MAPK), NF‐κB, gap junction, Wnt, ErbB, Jak‐STAT and apoptosis signalling pathways in CdCl_2_‐treated H9‐CMs (Figure 5J and Table S7).

### 3.8 P38 MAPK signalling pathway is critical to CIC in H9‐CMs

**Figure 6:**
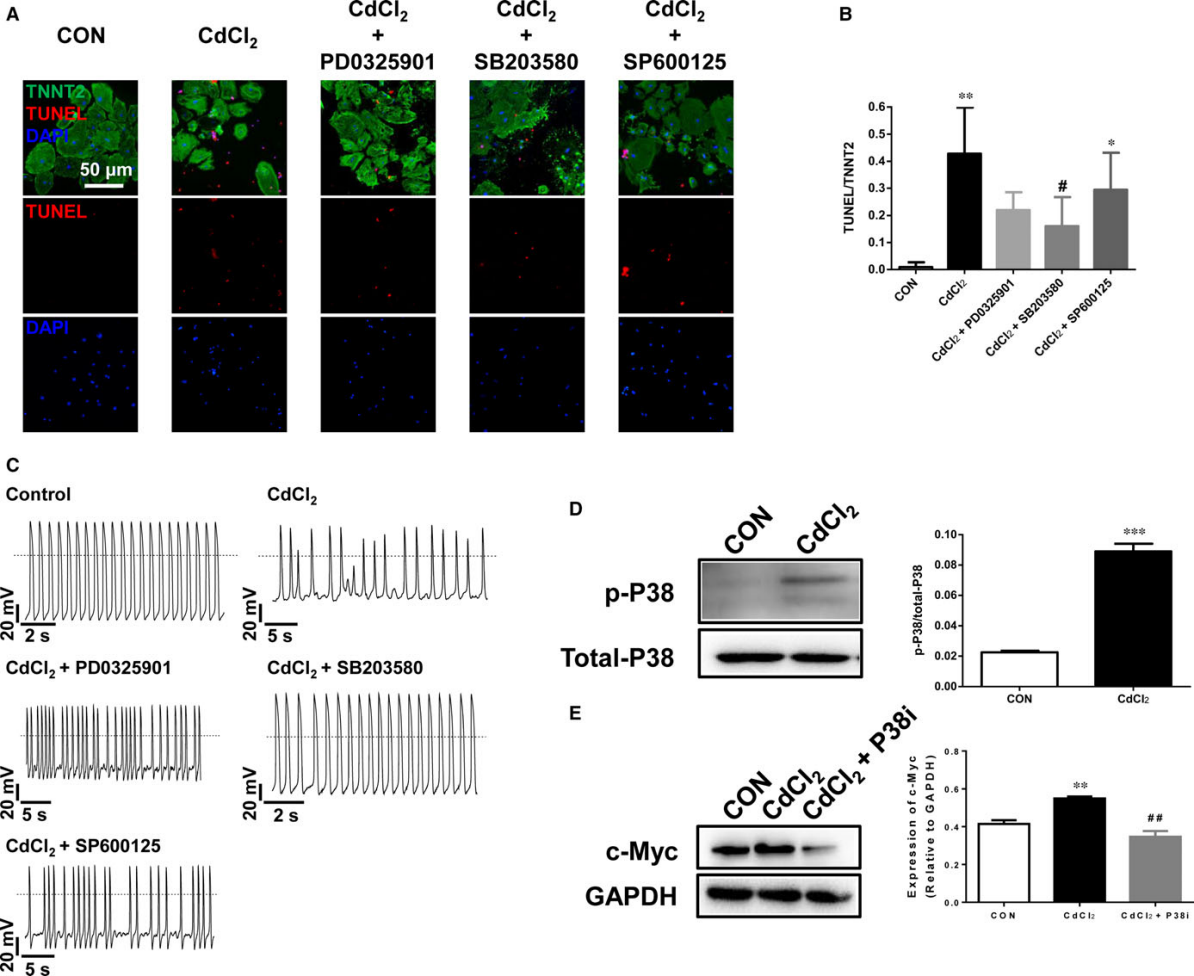
P38 MAPK signalling pathway is critical to CIC in H9‐CMs. A, Representative confocal images showing the rescuing effect of 30 μmol/L CdCl_2_‐induced apoptosis in H9‐CMs by specific blockers of ERK, P38 and JNK, respectively. Scale bar, 50 μm. B, Bar graph to compare the ratio of TUNEL/TNNT2 between different groups in A. *P < .05, **P < .01, when compared to control cells; ^#^ *P* < .05, when compared to CdCl_2_‐treated cells. C, Representative action potential tracings recorded from control H9‐CMs, H9‐CMs treated with 30 μmol/L CdCl_2_, H9‐CMs treated with 30 μmol/L CdCl_2_ and 1 μmol/L PD0325901 (ERKi), H9‐CMs treated with 30 μmol/L CdCl_2_ and 10 μmol/L SB203580 (P38i), H9‐CMs treated with 30 μmol/L CdCl_2_ and 10 μmol/L SP600125 (JNKi). D, Left panel, Western blot analysis of p‐P38 expression in control and 30 μmol/L CdCl_2_‐treated H9‐CMs; Right panel, bar graph to compare the p‐P38 expression between control and CdCl_2_‐treated cells. ***P < .001. E, Left panel, Western blot analysis of the c‐Myc expression in control H9‐CMs, H9‐CMs treated with 30 μmol/L CdCl_2_, H9‐CMs treated with 30 μmol/L CdCl_2_ and 10 μmol/L SB203580 (P38i); Right panel, bar graph to compare the c‐Myc expression between different groups. **P < .01, when compared to control cells; ^##^ *P* < .01, when compared to CdCl_2_‐treated cells

To relate changes in gene expression to functional consequences, we next investigated MAPK signalling pathway, which was associated with cadmium‐induced apoptosis of several cell types in previous studies and was dramatically up‐regulated in CdCl_2_‐treated H9‐CMs.^47^, ^48^, ^49^, ^50^, ^51^, ^52^, ^53^MAPK consists of extracellular signal‐regulated kinases (ERK), P38 and c‐Jun N‐terminal kinases (JNK) signalling pathways. We therefore test whether blockade of specific signalling pathway can reverse cadmium‐induced cellular phenotype. H9‐CMs were exposed to 30 μmol/L CdCl_2_ in the presence of PD0325901 (ERK‐specific inhibitor, ERKi), SB203580 (P38‐specific inhibitor, P38i) or SP600125 (JNK‐specific inhibitor, JNKi), respectively. In comparison with H9‐CMs treated with CdCl_2_ alone, addition of SB203580 attenuated the CdCl_2_‐induced apoptosis measured by TUNEL assay (Figure 6A,B). In contrast, PD0325901 or SP600125 showed negligible effect on rescuing the CdCl_2_‐induced cell apoptosis (Figure 6A,B). Consistently, SB203580 but not PD0325901 or SP600125, significantly alleviated CdCl_2_‐induced arrhythmias and irregular heartbeat phenotype in H9‐CMs, showing a normal action potential profile similar to control cells (Figures 6C and S11). We next performed Western blot to assess the expression of phosphorylated P38 (p‐P38) at the protein level, as well as expression of c‐Myc which is a downstream target of MAPKAPK2/MAPKAPK3 in P38 MAPK signalling pathway. CdCl_2_‐treated H9‐CMs exhibited significantly increased expression of p‐P38 and c‐Myc compared to control cells, indicating an activated P38 MAPK signalling pathway during cadmium induction (Figure 6D). However, addition of SB203580, selectively targeting MAPKAPK2 and MAPKAPK3, effectively attenuated increased expression of c‐Myc induced by CdCl_2_ treatment (Figure 6E). These data suggest that P38 MAPK signalling pathway is critical to CIC in H9‐CMs and suppression of P38 can protect CIC.

### 3.9 Protection of CIC by suppressing PI3K‐Akt signalling pathway in H9‐CMs

**Figure 7:**
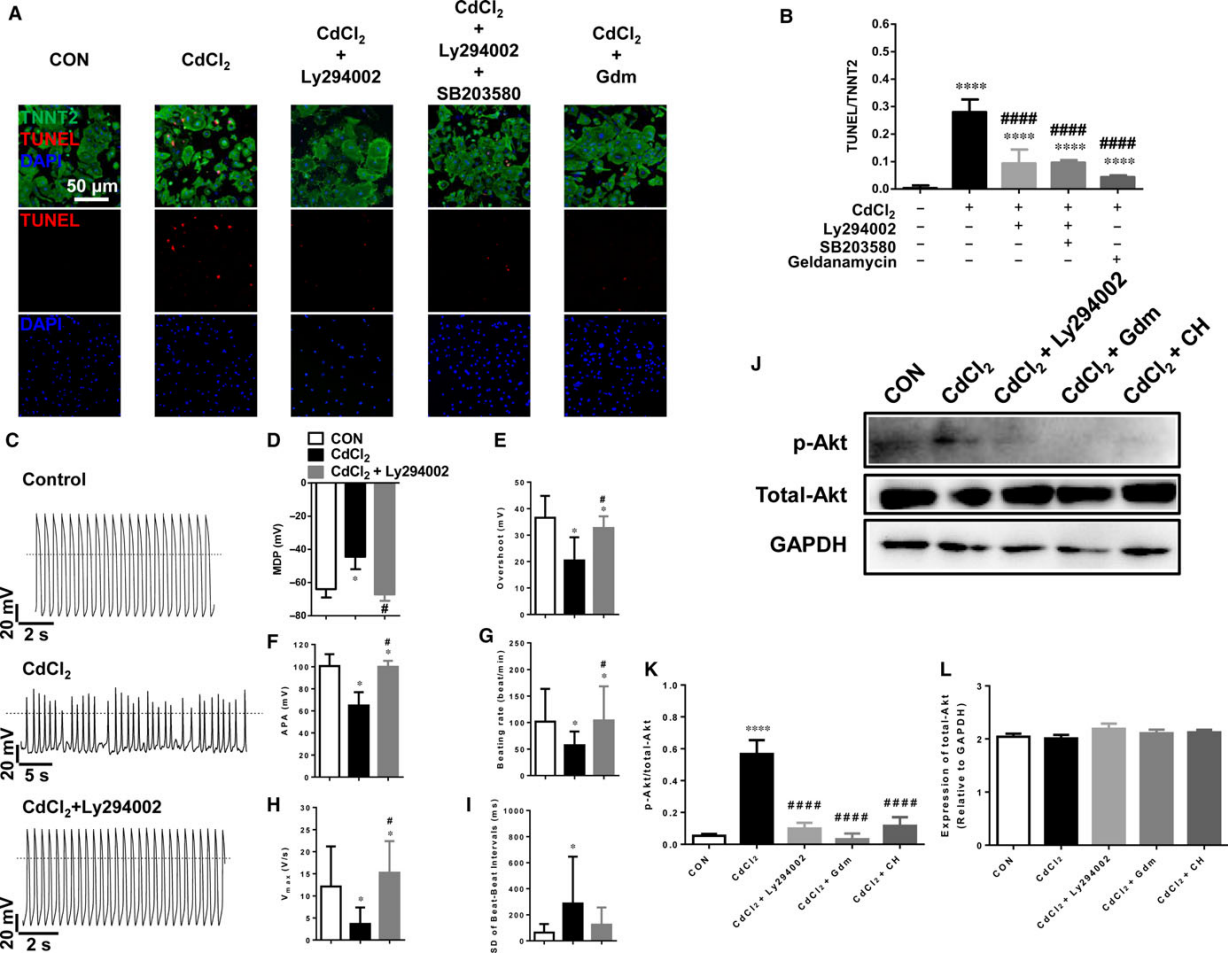
Protection of CIC by suppressing PI3K‐Akt signalling pathway in H9‐CMs. A, Representative confocal images showing the rescuing effect of 30 μmol/L CdCl_2_‐induced apoptosis in H9‐CMs by a specific PI3K/Akt blocker Ly294002 (25 μmol/L), Ly294002 and SB203580, and geldanamycin (0.5 μmol/L). Scale bar, 50 μm. B, Bar graph to compare the ratio of TUNEL/TNNT2 between different groups in A. ****P < .0001, when compared to control cells; ^####^ *P* < .0001, when compared to CdCl_2_‐treated cells. C, Representative action potential tracings recorded from control H9‐CMs, H9‐CMs treated with 30 μmol/L CdCl_2_, H9‐CMs treated with 30 μmol/L CdCl_2_ and 25 μmol/L Ly294002. D‐I, Bar graph to compare MDP, Overshoot, APA, Beating rate, *V*_max_ and SD of beat–beat Intervals between control H9‐CMs, H9‐CMs treated with CdCl_2_, and H9‐CMs treated with CdCl_2_ and Ly294002, respectively. *P < .05, when compared to control cells; ^#^ *P* < .05, when compared to CdCl_2_‐treated cells. J, Western blot analysis of p‐Akt expression in control H9‐CMs, H9‐CMs treated with 30 μmol/L CdCl_2_, H9‐CMs treated with 30 μmol/L CdCl_2_ and 25 μmol/L Ly294002, H9‐CMs treated with 30 μmol/L CdCl_2_ and 0.5 μmol/L geldanamycin, H9‐CMs treated with 30 μmol/L CdCl_2_ and 40 μg/mL CH. K, Bar graph to compare the p‐Akt expression between different groups in J. ****P < .0001, when compared to control cells; ^####^ *P* < .0001, when compared to CdCl_2_‐treated cells. L, Bar graph to compare the total Akt expression between different groups in J

Previous studies reported that PI3K/Akt signalling pathway was associated with cadmium‐induced apoptosis in glial cells and proximal tubular cells.^47^, ^61^ Our RNA‐Seq data showed HSP90 genes (HSP90B1 and HSP90AB1) were highly expressed in CdCl_2_‐treated H9‐CMs, which may enhance phosphorylation of Akt to induce cell apoptosis (Figures S12 and S13).^62^, ^63^ Above clues encouraged us to investigate whether PI3K‐Akt signalling pathway plays an important role in CIC. Strikingly, Ly294002, a specific PI3K/Akt inhibitor, effectively rescued 30 μmol/L CdCl_2_‐induced apoptosis in H9‐CMs, with a greater rescuing effect than P38‐specific inhibitor SB203580 (Figure 7A,B). However, addition of Ly294002 and SB203580 together did not give rise to a further rescuing effect compared to Ly294002 alone, suggesting that PI3K‐Akt and P38 MAPK pathways may share the same downstream target to regulate CIC in H9‐CMs (Figure 7A,B). Moreover, CdCl_2_‐induced cell apoptosis was significantly rescued by geldanamycin, a HSP90 inhibitor, indicating that up‐regulation of HSP90 is associated with CdCl_2_‐induced apoptosis (Figure 7A,B). In line with the TUNEL data, addition of Ly294002 effectively rescued 30 μmol/L CdCl_2_‐induced electrophysiological phenotype, showing a normal action potential profile similar to control cells (Figure 7C‐I). We further confirmed that phosphorylation of Akt (p‐Akt) was activated in 30 μmol/L CdCl_2_‐induced H9‐CMs, whereas addition of Ly294002 or geldanamycin effectively reversed the increased level of p‐Akt expression, suggesting that activated Akt is associated with cadmium‐induced apoptosis which is HSP90 dependent (Figure 7J,K). However, there was no significant difference of total Akt expression between different groups (Figure 7L). Interestingly, similar rescuing effect was also observed in cells treated with CH, confirming that elevated cellular ROS is associated with enhanced PI3K/Akt signalling in CdCl_2_‐treated H9‐CMs and may be a key mediator of CIC (Figure 7J,K). Taken together, these data suggest that cadmium‐induced elevation of HSP90 expression or ROS amount may activate PI3K‐Akt signalling pathway, which is critical to CIC in H9‐CMs and suppression of PI3K/Akt is sufficient to protect CIC.

## 4 DISCUSSION

Cardiovascular diseases rank No.1 morbidity and mortality worldwide. Cadmium, a highly ubiquitous toxic heavy metal, was shown to damage cardiovascular system, thus leading to cardiovascular diseases such as myocardial infarction, peripheral arterial disease, cardiomyopathy, hypertension and arteriosclerosis, stroke and heart failure.^17‐23^ Previous studies have shown that cadmium can induce apoptosis and is cardiotoxic in rat cardiomyocytes.^24‐30^ However, the mechanism by which cadmium causes the cardiotoxicity has not been studied in human cardiomyocytes at the cellular level. In this study, we show that, for the first time, hPSC‐CMs can serve as a unique tool to model cadmium‐induced cardiotoxicity in a dish, and this cell model recapitulates deleterious CIC phenotype.

Cardiomyocytes isolated from human heart are extremely difficult to obtain and usually do not survive in culture for very long, thus limiting their value as a tool for studying the cardiovascular diseases. Mouse models can recapitulate the human disease phenotype and have provided extensive insight into understand the underlying mechanisms. However, several differences exist between the mouse and human models. For example, the resting heart rate of mouse is about 6‐10‐fold faster than that of human. The Ca^2+^ handling and electrophysiological properties, ion channel expression and function, and cardiac development of mouse cardiomyocytes are different from those of human. Therefore, the species differences reduce the value of mouse models for understanding the cellular and molecular mechanisms underlying human cardiovascular diseases.

hPSC‐CMs are human cells and display many features of human adult ventricular CMs, offering a human‐based and physiology‐relevant cell source for disease modelling and drug screening. They can also be scaled up to reproducibly produce large cell numbers, which are likely the best currently‐available model to model disease phenotype and drug responses.^64‐70^

We treated H9‐CMs with escalating doses of CdCl_2_ (0.1‐100 μmol/L) for 24 hours, observed morphological changes and cell apoptosis in a dose‐dependent manner. With treatment of 30 μmol/L CdCl_2_ for 24 hours, H9‐CMs exhibited sarcomeric disorganization and disruption, abnormal ultrastructure with enlarged nucleocytoplasmic ratio and nuclear membrane shrinkage. In line with previous studies, we also observed significantly elevated ROS production in 30 μmol/L CdCl_2_‐treated H9‐CMs as compared to control cells which can be significantly reversed by an anti‐oxidant CH, suggesting that ROS‐based toxicity is involved in the increased apoptosis of cadmium‐treated H9‐CMs and the elevated ROS may serve as a key mediator to induce downstream signalling events leading to apoptosis and cell dysfunction of cardiomyocytes.^24,26‐29^

Effects of cadmium on the heart include effects of cardiac structure and integrity, as well as effects of cardiac conduction system.^23^ However, the in vitro electrophysiology of CMs exposed to cadmium has not been well studied yet. We observed that, when treated with 30 μmol/L CdCl_2_ for 24 hours, H9‐CMs exhibited cardiac arrhythmias including EADs and DADs, following with a series of electrophysiological changes including reduced depolarized MDP, reduced overshoot and APA, slower beating rate, slower depolarization rate and increased beat‐beat interval variability. The abnormal electrophysiological phenotype induced by cadmium can be rescued by inhibition of PI3K‐Akt or P38 MAPK signalling pathway. Concerted ion channels shape the cardiac action potential, thus control the electrophysiology and excitability in cardiac cells. We showed a distinct ion channel gene expression profile as well as reduced sodium and calcium currents in CdCl_2_‐treated H9‐CMs, suggesting that cells experienced cardiac ion channel remodelling in response to cadmium induction which may account for the altered electrophysiology and cardiac arrhythmias associated with PI3K/Akt and P38 MAPK.

Generation of purified H9‐CMs allows us to perform accurate RNA‐Seq analysis without contamination of other cell types. Compared to control H9‐CMs, CdCl_2_‐treated cells exhibited differential transcriptome profile. A large number of genes distinctly expressed in CdCl_2_‐treated cells, in which MT, HSP, GADD45 and DUSP genes were up‐regulated, whereas cardiac development and morphogenesis related genes were down‐regulated. Notably, the significantly elevated expression of numerous MT gene isoforms observed in 30 μmol/L CdCl_2_‐treated H9‐CMs suggests a cellular defensive mechanisms of CMs in response to cadmium induction. GO analysis revealed that genes were positively enriched in “regulation of cell death,” “regulation of apoptotic process,” “regulation of programmed cell death,” “regulation of response to stimulus” and “regulation of response to stress.” IPA identified up‐regulation of protein processing in MAPK, NF‐κB, gap junction, Wnt, ErbB, Jak‐STAT and apoptosis signalling pathways in CdCl_2_‐treated H9‐CMs. We further found that P38 MAPK inhibitor partially protected CdCl_2_‐induced cell apoptosis and abnormal electrophysiology, suggesting that activation of P38 MAPK signalling pathway involved in cadmium‐induced cardiotoxicity.

Previous studies have reported that PI3K/Akt signalling pathway is associated with cadmium‐induced apoptosis in different cell types.^47^, ^61^, ^71^, ^72^, ^73^, ^74^, ^75^ Notably, 3 studies have shown that activation of PI3K/Akt signalling pathway led to cadmium‐induced apoptosis in astrocytes, thyroid carcinoma cells and HK‐2 renal proximal tubular epithelial cells, respectively.^47^, ^71^, ^74^ Indeed, we identified in this study that PI3K/Akt inhibitor restored CdCl_2_‐induced apoptosis and abnormal electrophysiology to control H9‐CMs, supporting the sustained PI3K/Akt signalling pathway activation and Akt‐mediated cardiotoxicity induced by cadmium.

In summary, our results suggest that hPSC‐CMs can recapitulate the CIC in vitro. We found cadmium‐induced elevation of ROS amount, and subsequent ROS accumulation induced downstream signalling events including PI3K/Akt and P38 MAPK that led to reduced viability, apoptosis and cell dysfunction of cardiomyocytes. We identified PI3K/Akt as well as P38 MAPK signalling pathways played an important role in CIC. Our study provides a reliable model with cellular phenotype associated with CIC, which enhances discovery of new cardioprotective drugs by pinpointing the underlying molecular basis.

## CONFLICTS OF INTEREST

The authors confirm that there are no conflicts of interest.

## AUTHOR CONTRIBUTIONS

P.L. designed and supervised the study. J.S., X.W., D.Z., T.L., L.T., T.G. and J.S. performed the experiments and analysed data. P.L. wrote the manuscript.
