# Supplementary material for: Modelling cadmium‐induced cardiotoxicity using human pluripotent stem cell‐derived cardiomyocytes
Source: J Cell Mol Med. 2018 Jul 11;22(9):4221–35. doi: 10.1111/jcmm.13702 (PMC6111808; doi:10.1111/jcmm.13702)
Supplement: Supplementary file 1 [file JCMM-22-4221-s001.pdf]

SUPPLEMENTAL FIGURE LEGENDS

Supplemental Figure 1. Generation of H9 human embryonic stem cell-derived cardiomyocytes (H9-CMs). A. Typical morphology of undifferentiated H9 hESCs. Scale bar, 250 μm. **B.** Pluripotent staining of H9 hESCs using NANOG (Green), SSEA4 (Red), OCT4 (Green) and SOX2 (Red). DAPI indicates nuclear staining (Blue). Scale bar, 100 μm. **C.** Alkaline Phosphatase (AP) staining of H9 hESCs. Scale bar, 50 μm. **D.** Typical morphology of differentiated H9 hESC-derived cardiomyocytes (H9-CMs). Scale bar, 250 μm. **E.** FACS analysis of TNNT2-positive cells. **F.** Immunofluorescent staining of H9-CMs using cardiac-specific markers TNNT2 (Green) and α-actinin (Red). DAPI indicates nuclear staining (Blue). Scale bar, 1 μm.

Supplemental Figure 2. Enlarged view of time-lapse live cell imaging of control untreated H9-CMs at 0 h, 6 h, 17 h, 20 h and 23 h.

Supplemental Figure 3. Enlarged view of time-lapse live cell imaging of H9-CMs treated with 30 μM $CdCl_2$ at 0 h, 6 h, 17 h, 20 h and 23 h.

Supplemental Figure 4. Increased Caspase-3 expression and activity in $CdCl_2$-treated H9-CMs. A. Western blot analysis of the Caspase-3 expression in control H9-CMs, H9-CMs treated with 30 μM $CdCl_2$, H9-CMs treated with 30 μM $CdCl_2$ and 20 μM Z-VAD-FMK. **B.** Bar graph to compare the Caspase-3 expression between different groups. $^{****}P < 0.0001$, when compared to control cells; $^{\#\#\#\#}P < 0.0001$, when compared to $CdCl_2$-treated cells. **C.** Bar graph to compare the Caspase-3 activity in control H9-CMs, H9-CMs treated with 30 μM $CdCl_2$, H9-CMs

treated with 30 μM $CdCl_2$ and 20 μM Z-VAD-FMK. $^{***}P < 0.001$, when compared to control cells; $^{###}P < 0.001$, when compared to $CdCl_2$-treated cells. **D.** Representative confocal images showing the rescuing effect of 30 μM $CdCl_2$-induced apoptosis in H9-CMs by 20 μM Z-VAD-FMK. Scale bar, 50 μm. **E.** Bar graph to compare the ratio of TUNEL/TNNT2 between different groups in **D**. $^{****}P < 0.0001$, when compared to control cells; $^{####}P < 0.0001$, when compared to $CdCl_2$-treated cells. **F.** Bar graph to compare the cell viability in control H9-CMs, H9-CMs treated with 30 μM $CdCl_2$, H9-CMs treated with 30 μM $CdCl_2$ and 20 μM Z-VAD-FMK. $^{***}P < 0.001$, $^{****}P < 0.0001$, when compared to control cells; $^{##}P < 0.01$, when compared to $CdCl_2$-treated cells.

Supplemental Figure 5. Cadmium-induced elevated ROS in H9-CMs. A. Bar graph to compare the cellular ROS amount in control H9-CMs, H9-CMs treated with 30 μM $CdCl_2$, H9-CMs treated with 30 μM $CdCl_2$ and 40 μg/ml Catechin Hydrate (CH). $^{**}P < 0.01$, $^{****}P < 0.0001$, when compared to control cells; $^{###}P < 0.0001$, when compared to $CdCl_2$-treated cells. **B.** Bar graph to compare the cell viability in control H9-CMs, H9-CMs treated with 30 μM $CdCl_2$, H9-CMs treated with 30 μM $CdCl_2$ and 40 μg/ml CH. $^{***}P < 0.001$, $^{****}P < 0.0001$, when compared to control cells; $^{##}P < 0.01$, when compared to $CdCl_2$-treated cells. **C.** Representative confocal images showing the rescuing effect of 30 μM $CdCl_2$-induced apoptosis in H9-CMs by 40 μg/ml CH. Scale bar, 50 μm. **D.** Bar graph to compare the ratio of TUNEL/TNNT2 between different groups in **C**. $^{****}P < 0.0001$, when compared to control cells; $^{####}P < 0.0001$, when compared to $CdCl_2$-treated cells.

Supplemental Figure 6. Immunofluorescent staining of H9-CMs using cardiac-specific markers TNNT2 (Green) and α-actinin (Red). DAPI indicates nuclear staining (Blue). Scale bar, 1 μm.

Supplemental Figure 7. Transmission electron microscopy images of control and $CdCl_2$-treated H9-CMs at 5900×, 11500× and 26500× magnification, respectively.

Supplemental Figure 8. A. Voltage protocol used to record steady-state activation of sodium currents from H9-CMs. **B.** Voltage protocol used to record steady-state inactivation of sodium currents from H9-CMs. **C.** Voltage protocol used to record steady-state activation of calcium currents from H9-CMs. **D.** Voltage protocol used to record steady-state inactivation of calcium currents from H9-CMs.

Supplemental Figure 9. A. Venn diagram of differential expressed genes (DEGs) in three comparisons (Con1 vs. Cd1, Con2 vs. Cd2, Con3 vs. Cd3). **B.** Bar graph to compare DEG numbers in three comparisons (Con1 vs. Cd1, Con2 vs. Cd2, Con3 vs. Cd3). Con denotes Control and Cd denotes $CdCl_2$.

Supplemental Figure 10. Gene ontology (GO) analysis of DEGs between control and $CdCl_2$-treated H9-CMs.

Supplemental Figure 11. Bar graph to compare MDP **(9A)**, Overshoot **(9B)**, APA **(9C)**, the Beating rate **(9D)**, V_{max} **(9E)** and the SD of Beat-Beat Intervals **(9F)** between control H9-CMs, H9-CMs treated with $CdCl_2$, H9-CMs treated with $CdCl_2$ and PD0325901 (ERKi), H9-CMs treated with $CdCl_2$ and SB203580 (P38i), and H9-CMs treated with $CdCl_2$ and SP600125 (JNKi). $^{*}P< 0.05$, when compared to control cells; $^{\#}P< 0.05$, when compared to $CdCl_2$-treated cells.

Supplemental Figure 12. Bar graph to compare the FPKM values of HSP90.

Supplemental Figure 13. Schematic representation of HSP90 regulation of Akt.

Supplemental Figure 14. A. Western blot analysis of p-Akt expression in control H9-CMs, H9-CMs treated with 30 μM $CdCl_2$ and H9-CMs treated with 30 μM $CdCl_2$ and 25 μM Ly294002. **B.** Bar graph to compare the p-Akt expression between different groups in **A**. ****P*< 0.001, when compared to control cells; $^{\#\#}$*P*< 0.01, when compared to $CdCl_2$-treated cells.

SUPPLEMENTAL VIDEO LEGENDS

Supplemental Video 1. Contracting monolayer H9-CMs.

Supplemental Video 2. Contracting digested H9-CMs.

Supplemental Video 3. Tracking morphological changes of beating clusters of H9-CMs with different doses of $CdCl_2$ treatment for 24 h, 0 μM.

Supplemental Video 4. Tracking morphological changes of beating clusters of H9-CMs with different doses of $CdCl_2$ treatment for 24 h, 30 μM.

Supplemental Video 5. Tracking morphological changes of beating clusters of H9-CMs with different doses of $CdCl_2$ treatment for 24 h, 100 μM.

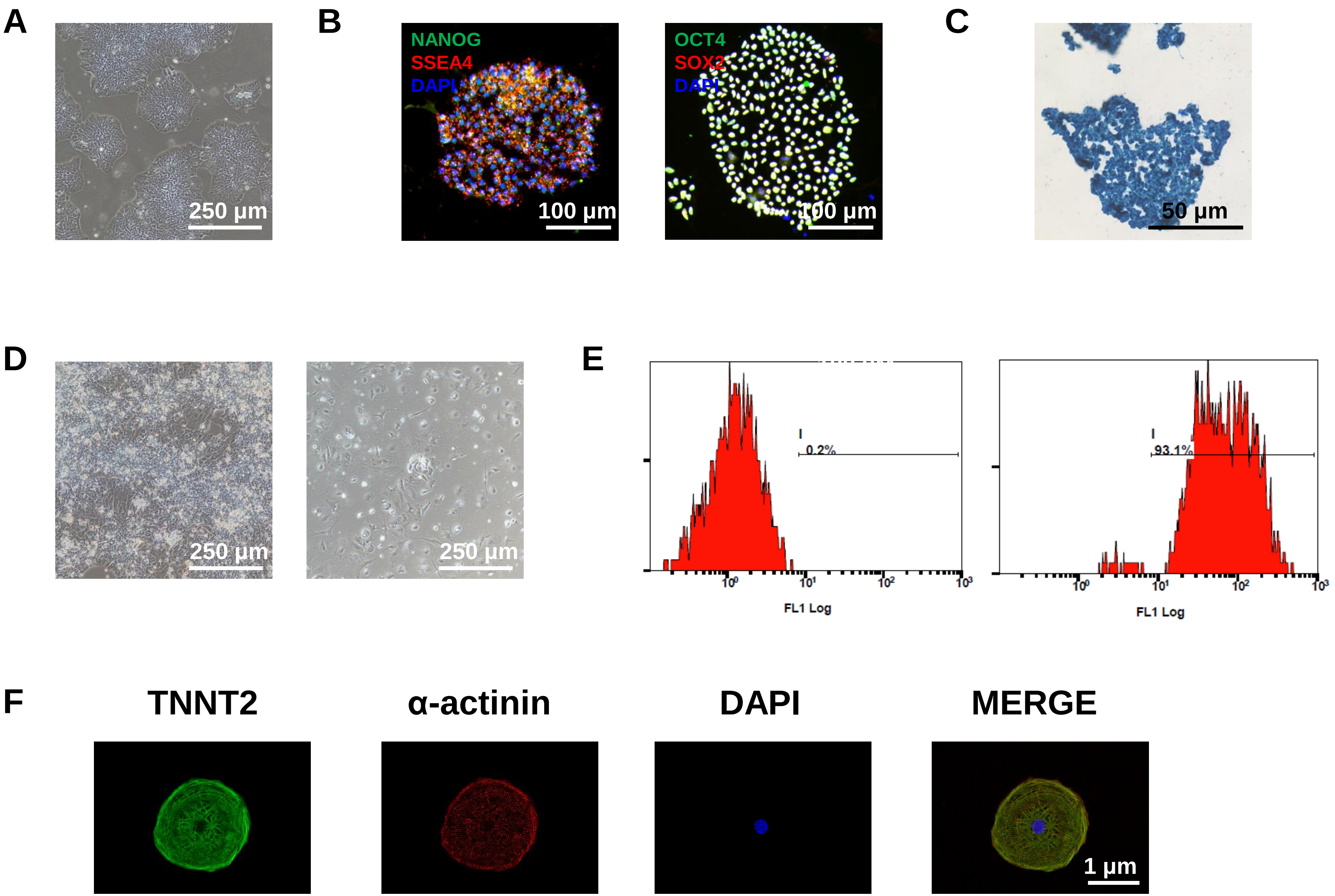

Supplemental Figure 1

Time-lapse live cell imaging in control untreated H9-CMs for 24 h

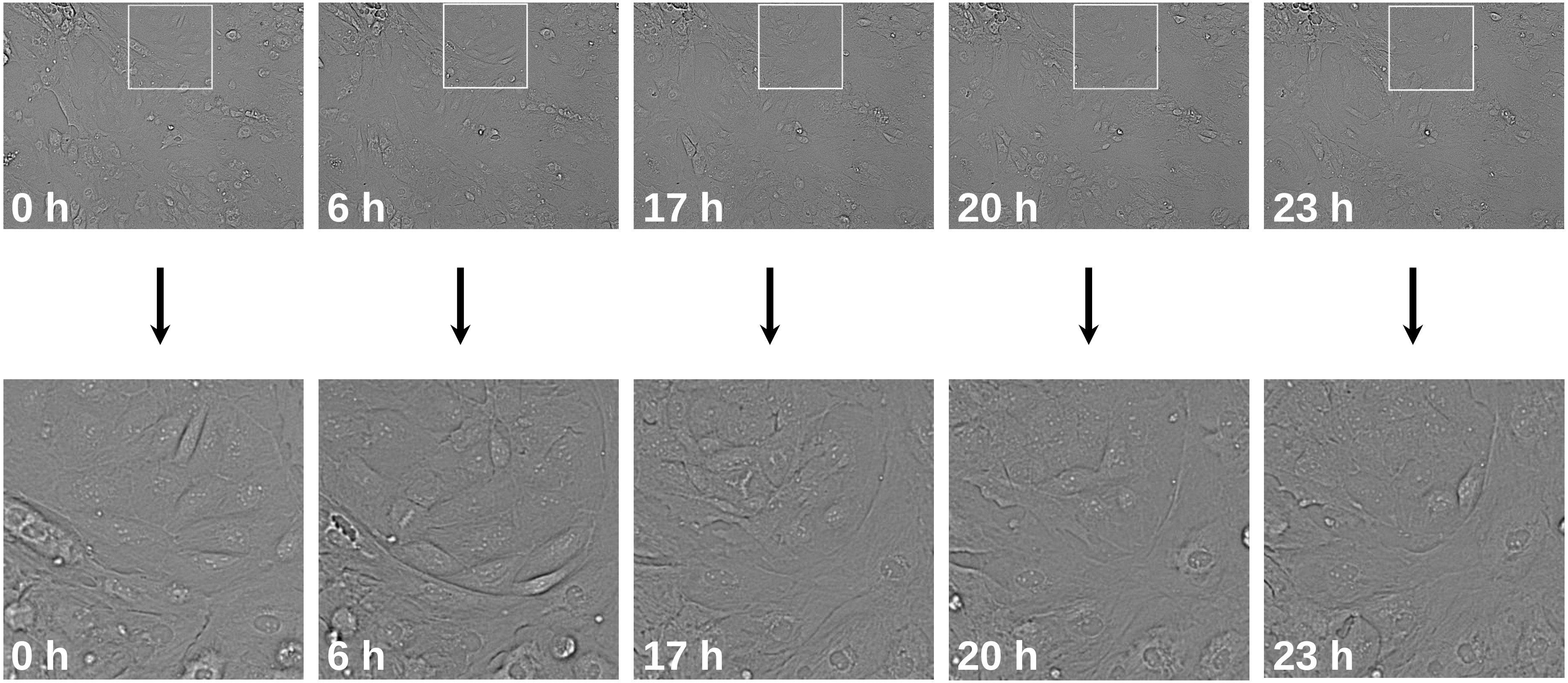

Supplemental Figure 2

Time-lapse live cell imaging in H9-CMs with 30 µM $CdCl_2$ for 24 h

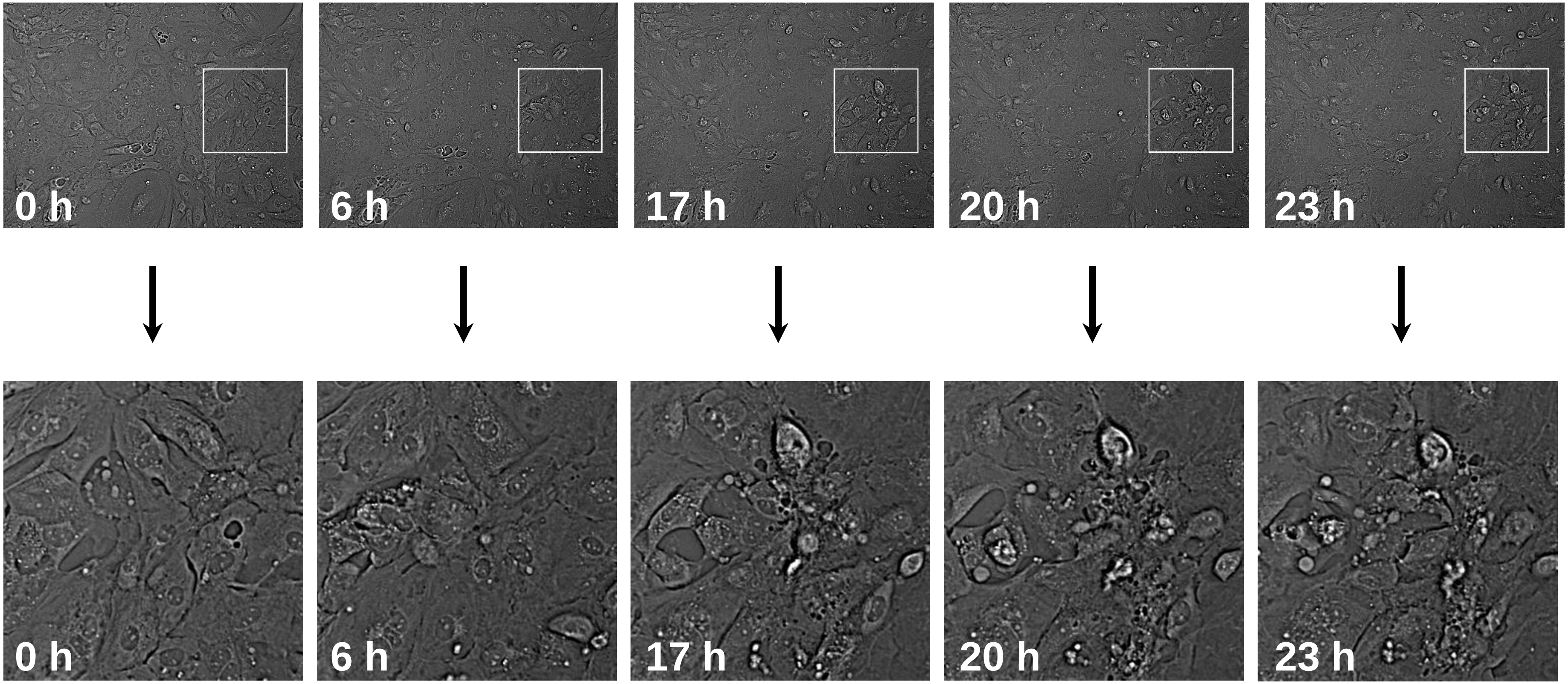

Supplemental Figure 3

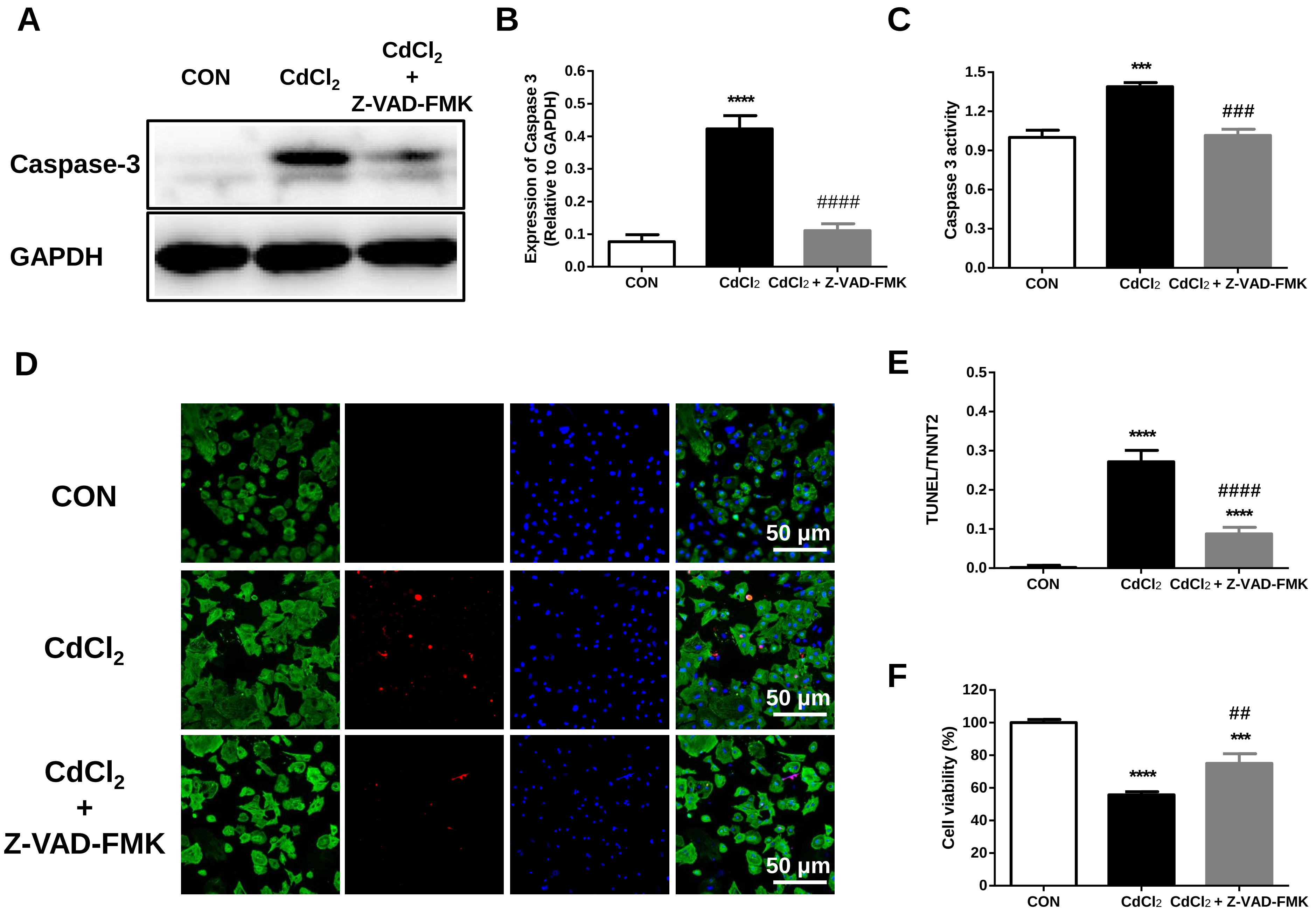

Supplemental Figure 4

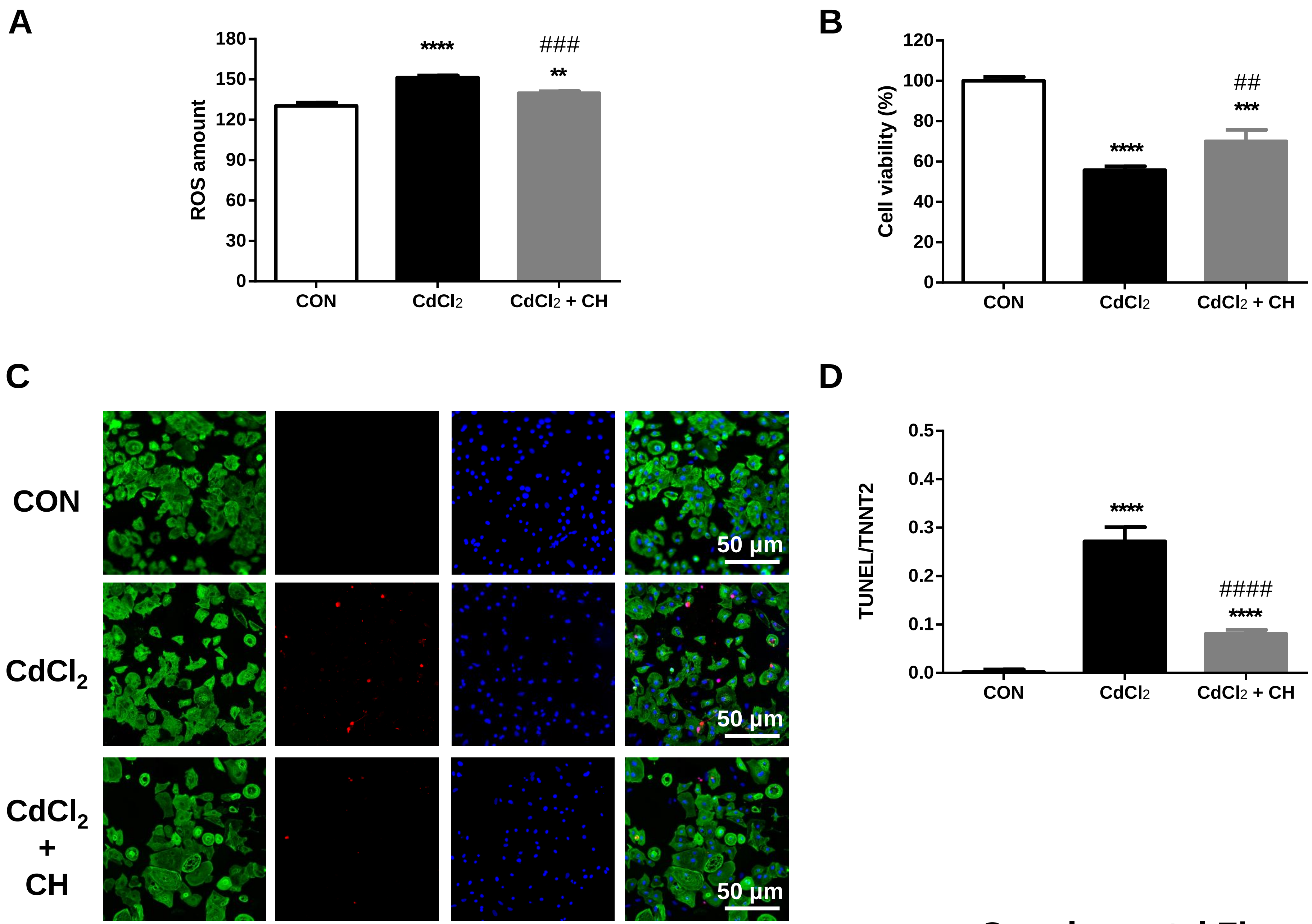

Supplemental Figure 5

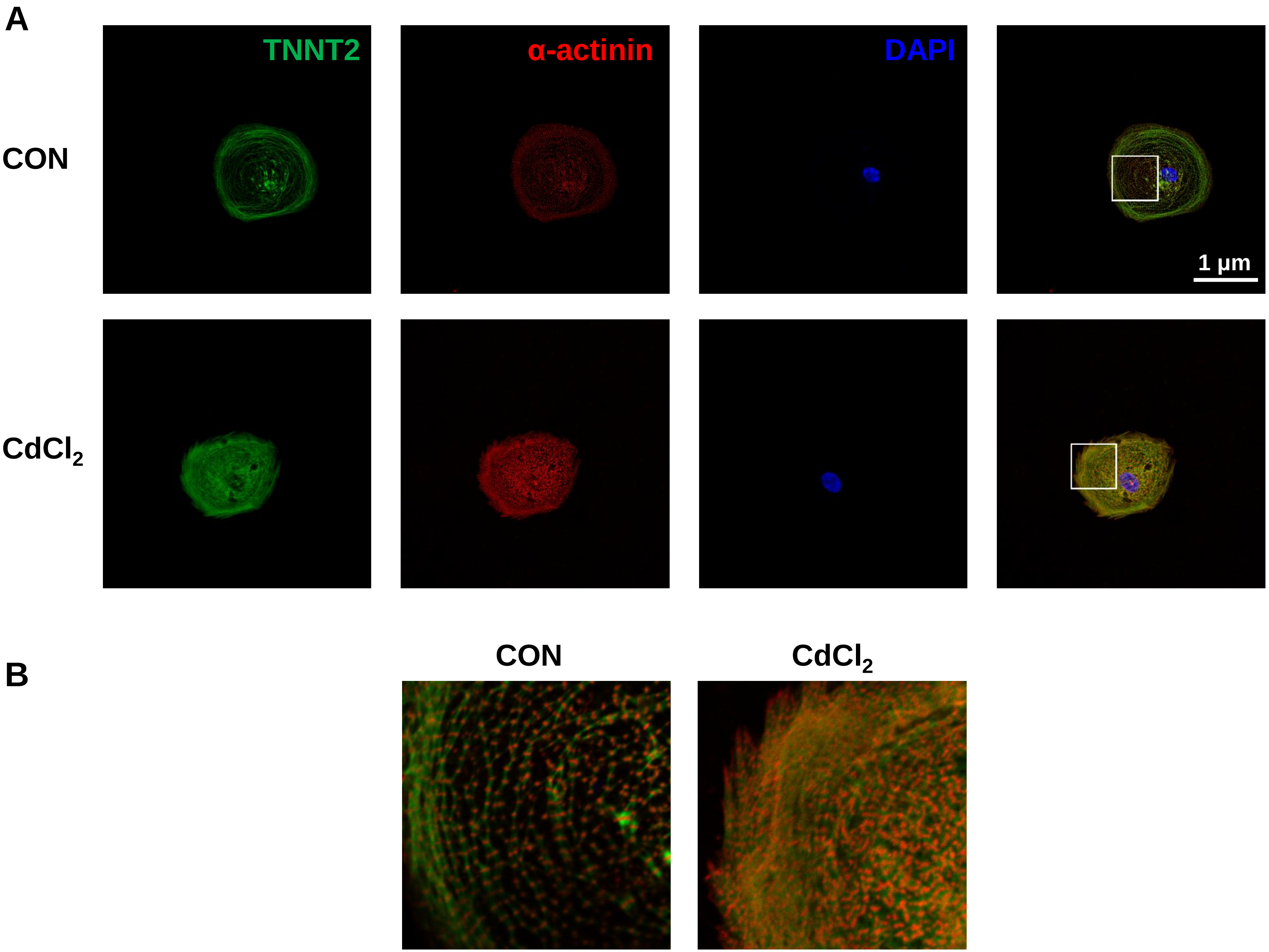

Supplemental Figure 6

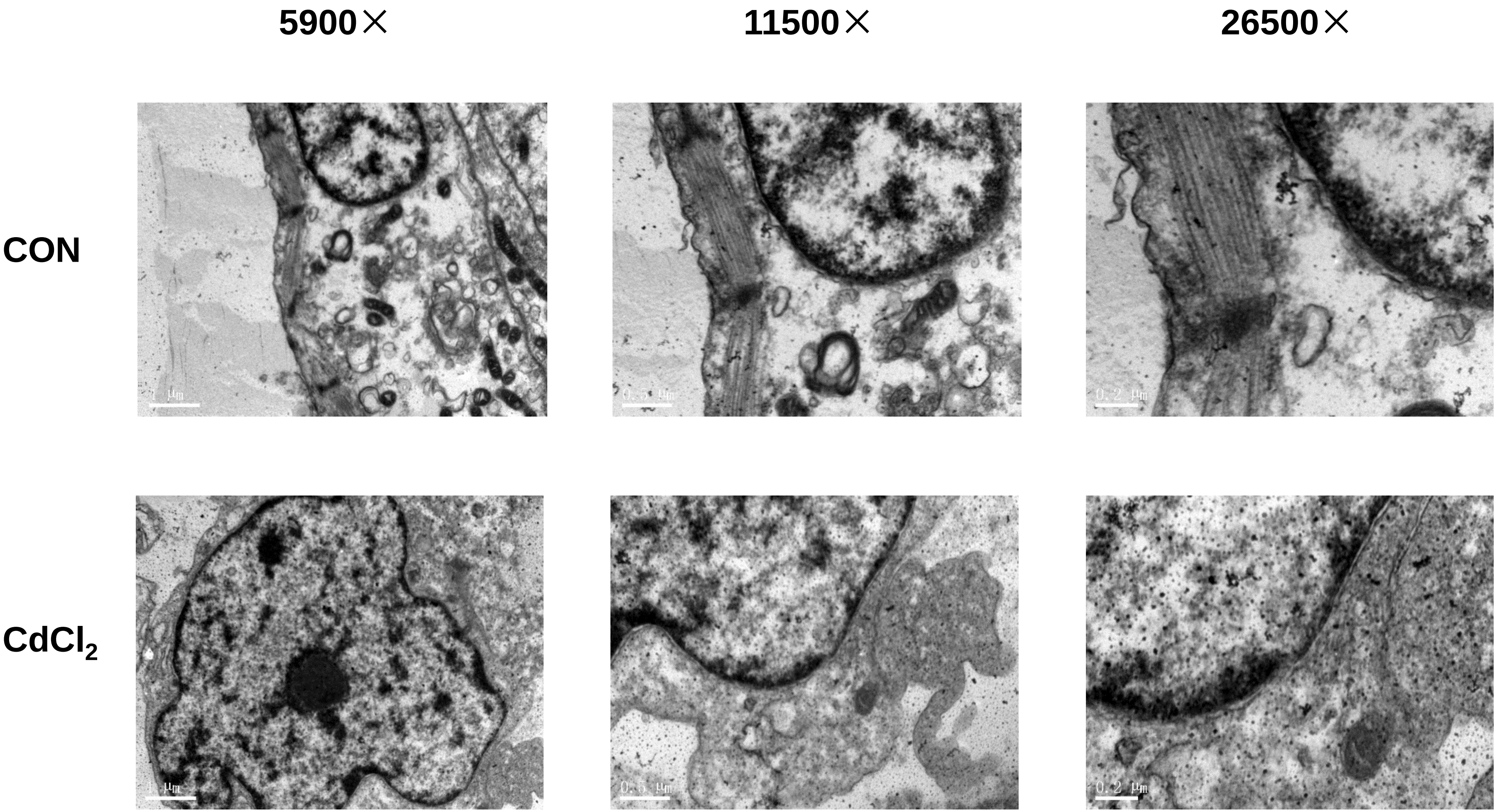

Supplemental Figure 7

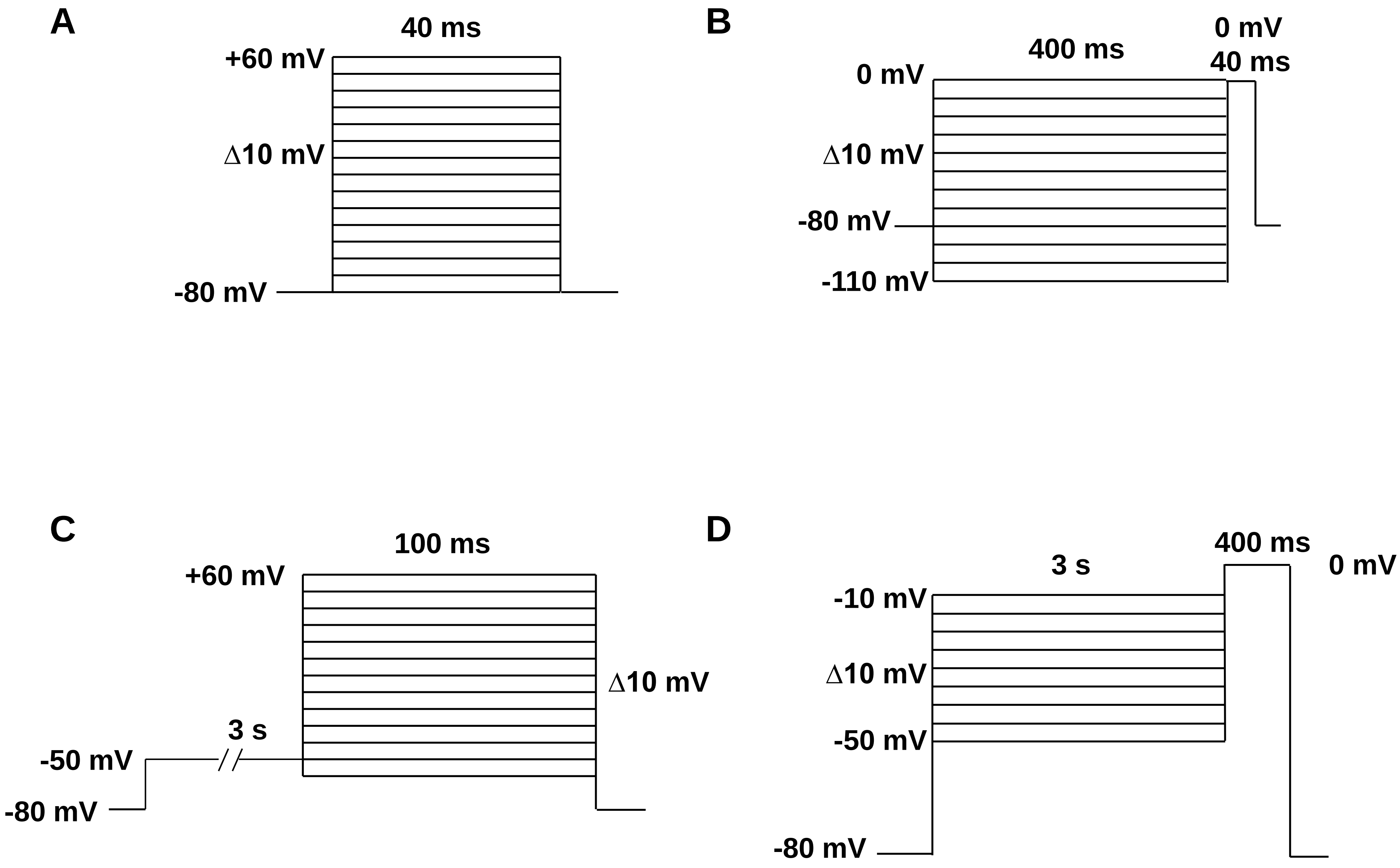

Supplemental Figure 8

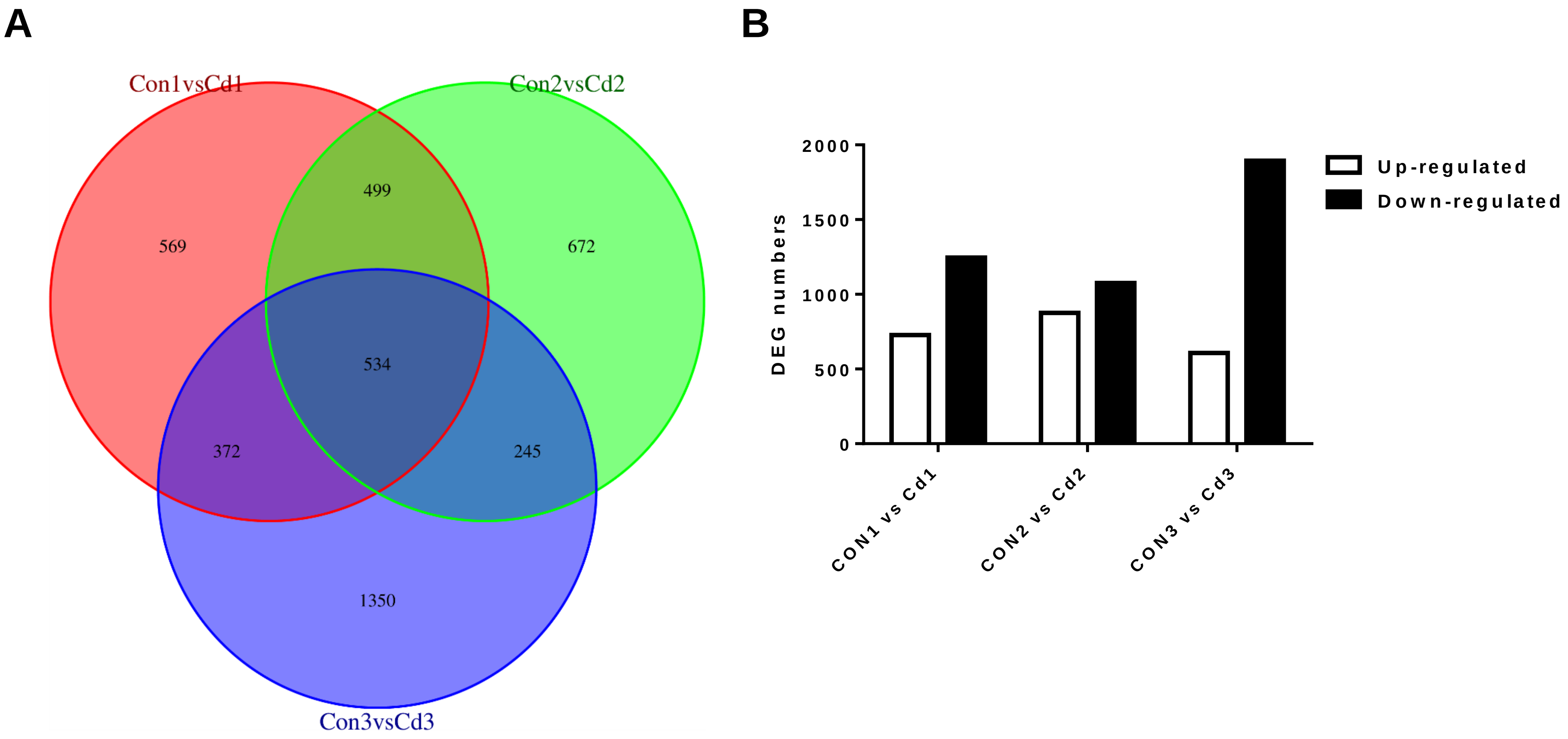

Supplemental Figure 9

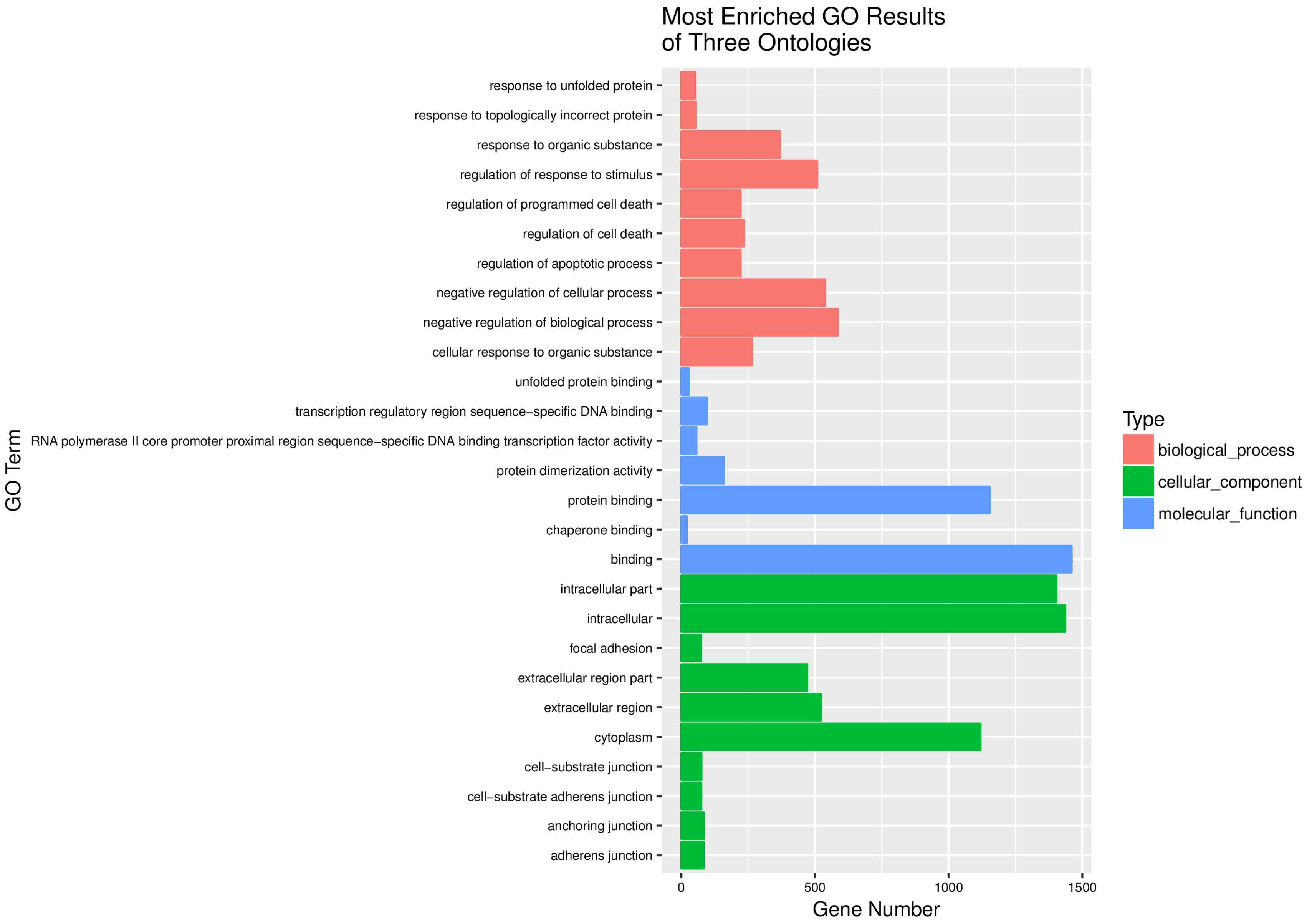

Supplemental Figure 10

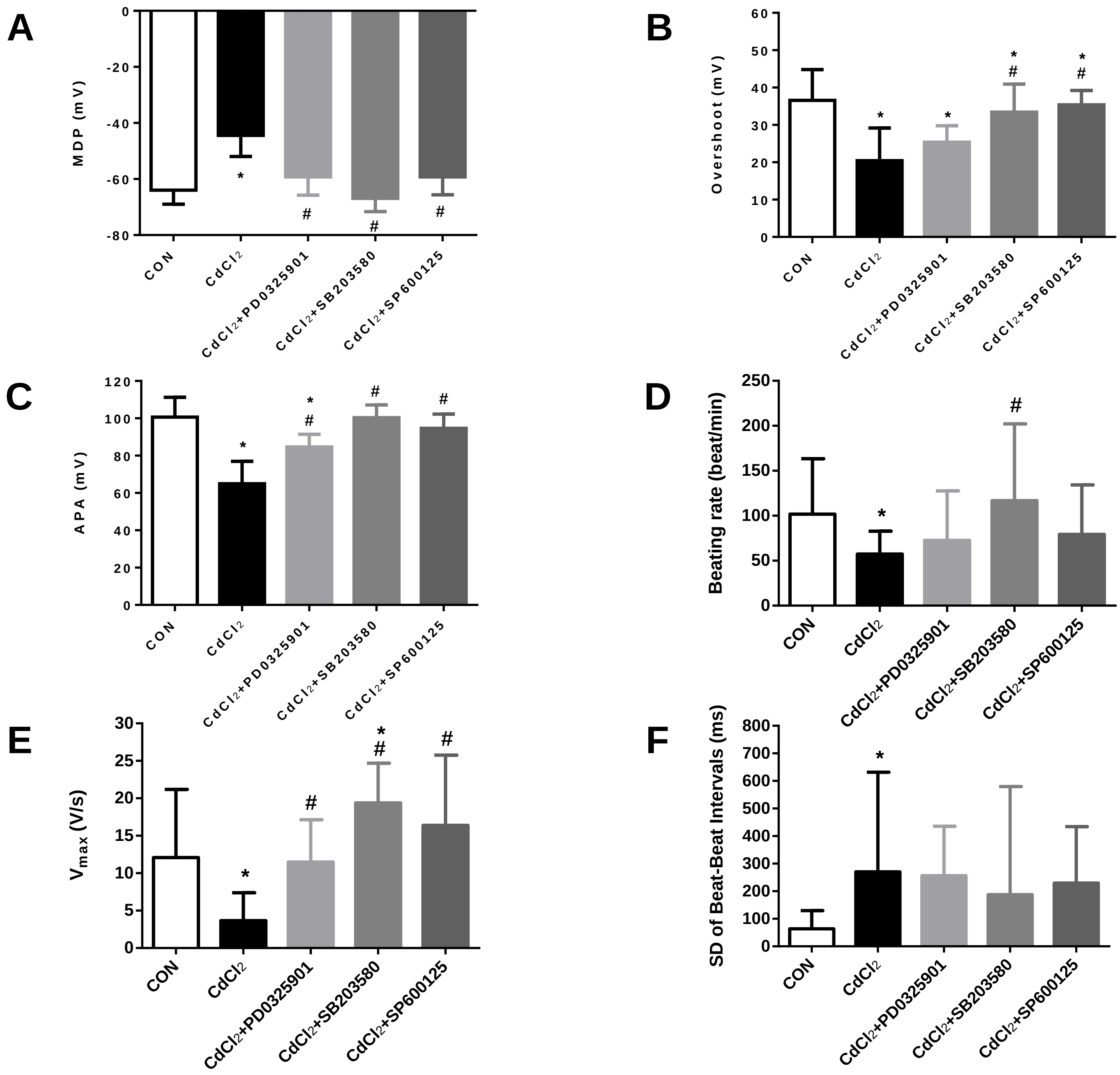

Supplemental Figure 11

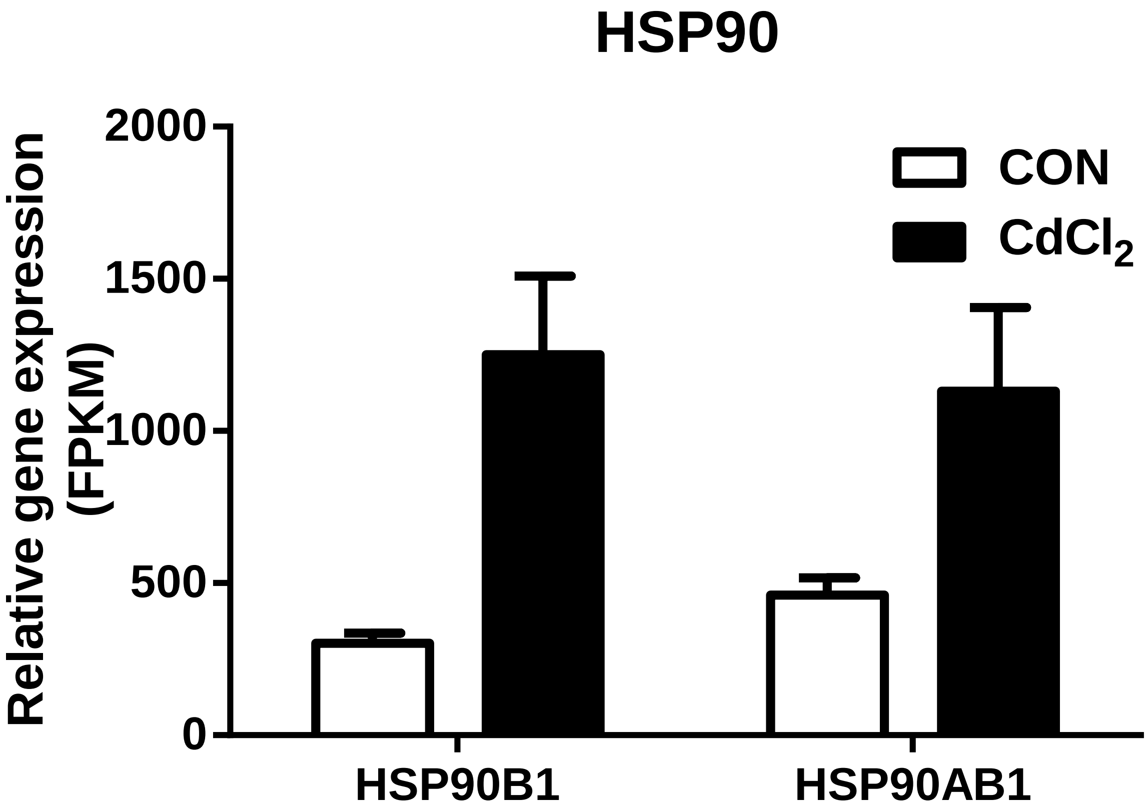

Supplemental Figure 12

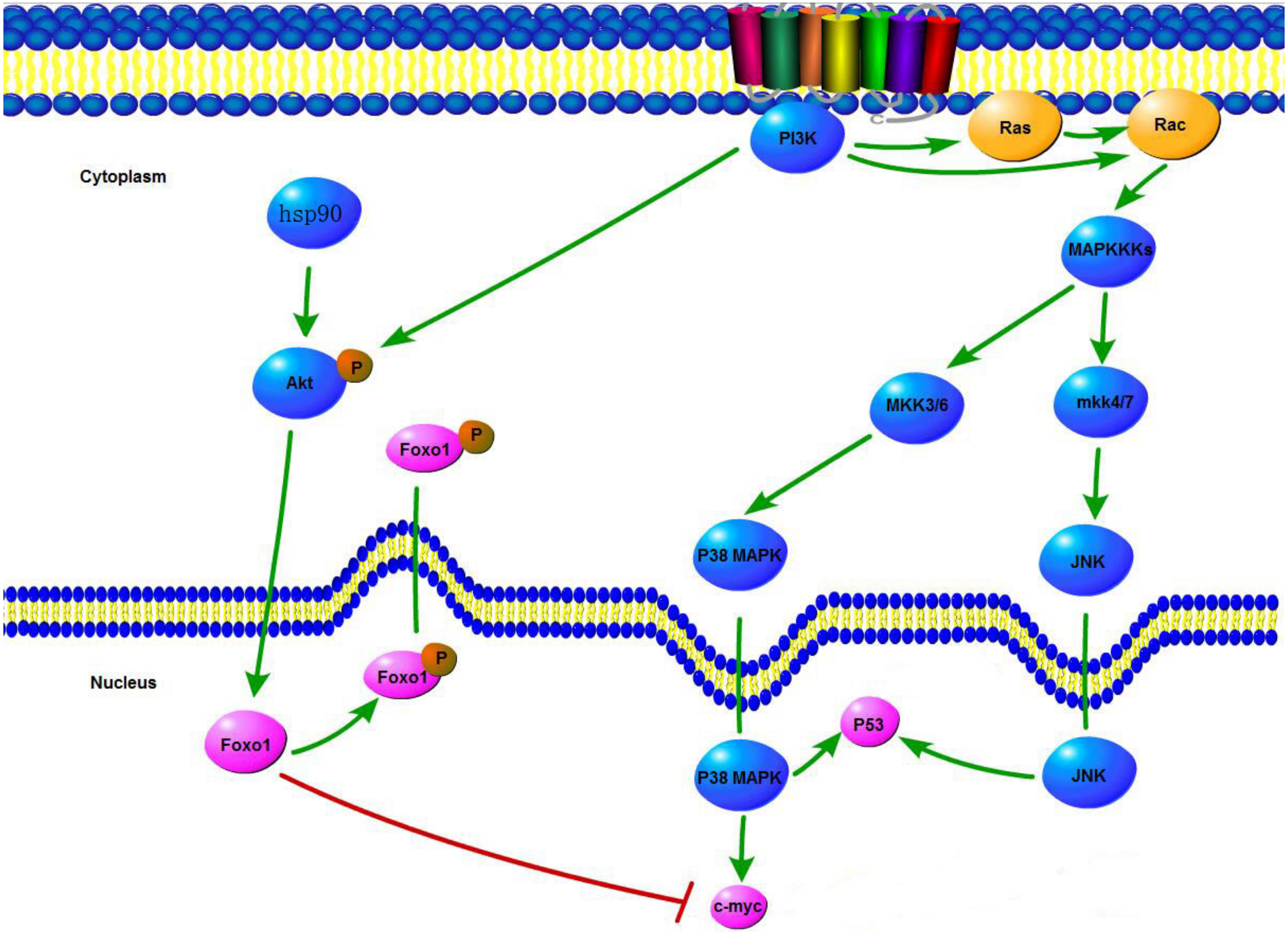

Supplemental Figure 13

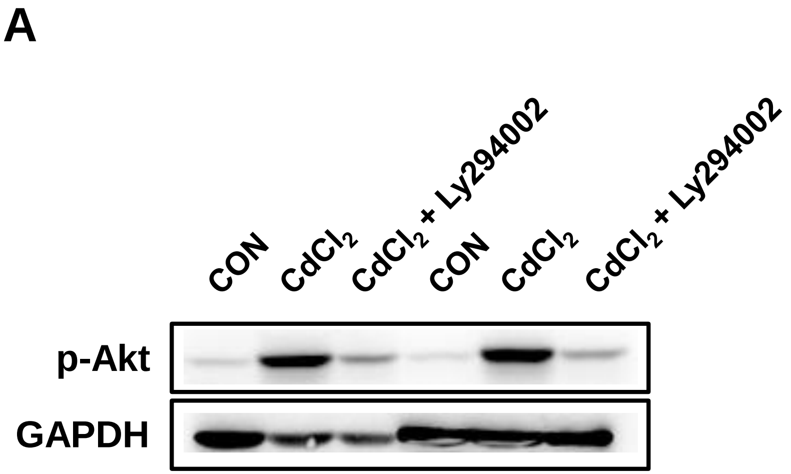

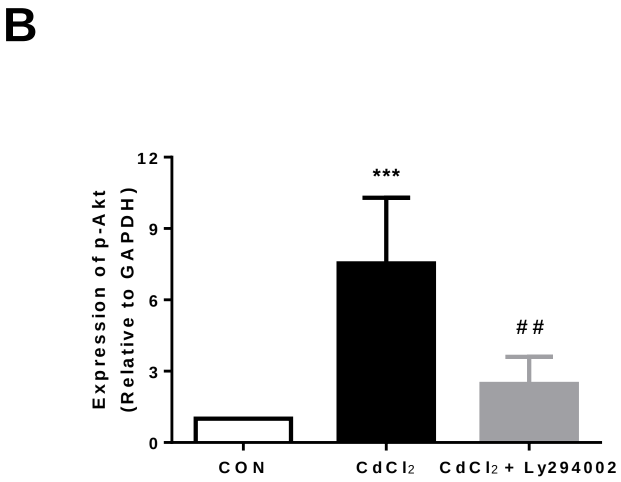

Supplemental Figure 14

Supplemental Table 1. Panel of ion channel genes used for qPCR

Gene Names	Forward	Reverse	Gene Description
GAPDH	GGTCGGAGTCAACGGATTTG	CGGTGCCATGGAATTTGCC	Glyceraldehyde-3-phosphate dehydrogenase
SCN5A	TCTCTATGGCAATCCACCCCA	GAGGACATACAAGGCGTTGGT	Sodium voltage-gated channel alpha subunit 5
KCND3	AGCTGATTGTCCTCAACGTGA	TCGTCGTCGTAGGCAGAGAT	Potassium voltage-gated channel subfamily D member 3
CACNA1C	TGATTCCAACGCCACCAATTC	GAGGAGTCCATAGGCGATTACT	Calcium voltage-gated channel subunit alpha1 C
KCNQ1	CGCCTGAACCGAGTAGAAGA	TGAAGCATGTCGGTGATGAG	Potassium voltage-gated channel subfamily Q member 1
KCNH2	CACCGCCCTGTACTTCATCT	AGGCCTTGCATACAGGTTCA	Potassium voltage-gated channel subfamily H member 2
KCNJ2	GTGCGAACCAACCGCTACA	CCAGCGAATGTCCACACAC	Potassium voltage-gated channel subfamily J member 2
KCNJ3	CCTGGCTTTTCATGGCGTC	GCAAGGCGTGTAGTTACCG	Potassium voltage-gated channel subfamily J member 3
KCNJ5	GCTGGCGATTCTAGGAATGC	TCTGTGGCAATGGGGACATAA	Potassium voltage-gated channel subfamily J member 5
KCNJ11	ACAGCCCACTCTACGACCT	CACTTTGACGGTGTTGCCA	Potassium voltage-gated channel subfamily J member 11
HCN2	ATCGTGTTCAACGTGGTCTCG	TGTTGTCCTCGATCACAATGC	Hyperpolarization activated cyclic nucleotide gated potassium and sodium channel 2
HCN4	GAACAGGAGAGGGTCAAGTCG	CATTGAAGACAATCCAGGGTGT	Hyperpolarization activated cyclic nucleotide gated potassium and sodium channel 4

Supplemental Table 2. Panel of compounds used in this study

Name of Compound	Stock Concentration	Working Concentration	Compound Description
PD0325901	1 mM	1 μM	ERK MAPK inhibitor
SB203580	10 mM	10 μM	P38 MAPK inhibitor
SP600125	10 mM	10 μM	JNK MAPK inhibitor
Ly294002	50 mM	25 μM	PI3K-Akt inhibitor
Z-VAD-FMK	20 mM	20 μM	Caspase inhibitor
Catechin Hydrate	50 mg/ml	40 μg/ml	Anti-oxidant
Geldanamycin	10 mM	0.5 μM	HSP90 inhibitor

Supplemental Table 3. Summary of action potential parameters in H9-CMs

	MDP (mV)	Overshoot (mV)	APA (mV)	APD_{90} (ms)	Beating Rate (Beats/min)	V_{max} (V/s)	SD of Beat-Beat Intervals (ms)
Control	-63.97 ±0.79	36.59 ±1.30	100.60 ±1.68	266.90 ±18.91	101.70 ±9.77	12.10 ±1.44	63.86 ±10.39
$CdCl_2$	-44.45 ±1.01*	20.42 ±1.17*	64.91 ±1.61*	289.9 ±15.58	57.40 ±3.40*	3.67 ±0.50*	287.80 ±47.99*
$CdCl_2$/PD0325901	-59.24 ±2.06#	25.35 ±1.40*	84.63 ±2.15*#	183.60 ±25.91	72.55 ±17.41	11.49 ±1.79#	256.40 ±56.60
$CdCl_2$/SB203580	-66.89 ±1.37#	33.44 ±2.15*#	100.30 ±1.98#	219.7 ±27.70	116.70 ±24.69#	19.38 ±1.53*#	187.60 ±113.10
$CdCl_2$/SP600125	-59.26 ±2.13#	35.40 ±1.27*#	94.66 ±2.64#	279.6 ±31.43	79.37 ±18.28	16.38 ±3.13#	229.60 ±68.21
$CdCl_2$/Ly294002	-67.12 ±1.22#	32.71 ±1.39*#	99.93 ±1.69#	188.5 ±23.01	104.10 ±20.33#	15.26 ±2.26#	124.50 ±41.79

* Compared to control H9-CMs

# Compared to $CdCl_2$-treated H9-CMs

Supplemental Table 4. Summary of sodium channel currents in H9-CMs

	Peak Current Density at -20 mV (pA/pF)	Cell Capacitance (pF)	Steady-state Activation		Steady-state Inactivation	
			$V_{1/2}$ (mV)	k	$V_{1/2}$ (mV)	k
Control	-180.67 ± 7.85	21.59 ± 1.70	-35.20 ± 0.93	2.90 ± 0.15	-68.36 ± 1.00	6.95 ± 0.29
$CdCl_2$	-75.20 ± 4.05*	23.86 ± 2.12	-36.07 ± 1.17	3.37 ± 0.22	-72.75 ± 1.71	7.04 ± 0.16

Supplemental Table 5. Summary of calcium channel currents in H9-CMs

	Peak Current Density at 0 mV	Cell Capacitance	Steady-state Activation		Steady-state Inactivation	
			$V_{1/2}$	k	$V_{1/2}$	k
	(pA/pF)	(pF)	(mV)		(mV)	
Control	-15.90 ± 1.36	46.78 ± 3.37	-10.24 ± 1.04	6.63 ± 0.37	-37.01 ± 1.71	6.23 ± 0.32
$CdCl_2$	-3.67 ± 0.53*	44.88 ± 3.63	-7.20 ± 1.20	7.26 ± 0.25	-39.94 ± 2.67	8.30 ± 0.98

Supplemental Table 6. Summary of GO analysis in $CdCl_2$-treated H9-CMs

Number	GO ID	GO Terms	Q Value	Total number of Genes
1	GO:0010033	Response to organic substance	2.18E-16	369
2	GO:0035966	Response to topologically incorrect protein	1.58E-12	54
3	GO:0006986	Response to unfolded protein	5.88E-12	51
4	GO:0048519	Negative regulation of biological process	1.06E-11	586
5	GO:0010941	Regulation of cell death	8.79E-11	235
6	GO:0042981	Regulation of apoptotic process	1.01E-10	222
7	GO:0043067	Regulation of programmed cell death	2.41E-10	222
8	GO:0048583	Regulation of response to stimulus	3.52E-10	509
9	GO:0071310	Cellular response to organic substance	6.72E-10	265
10	GO:0048523	Negative regulation of cellular process	8.50E-10	538
11	GO:0048518	Positive regulation of biological process	9.11E-10	679
12	GO:0048522	Positive regulation of cellular process	1.27E-09	593
13	GO:0007275	Multicellular organismal development	1.32E-09	566
14	GO:1901700	Response to oxygen-containing compound	1.59E-09	215
15	GO:0031323	Regulation of cellular metabolic process	1.90E-09	718
16	GO:0044767	Single-organism developmental process	2.88E-09	634
17	GO:0019222	Regulation of metabolic process	3.09E-09	805
18	GO:0032502	Developmental process	3.79E-09	642
19	GO:0070887	Cellular response to chemical stimulus	7.58E-09	307
20	GO:0080134	Regulation of response to stress	8.51E-09	218
21	GO:0010646	Regulation of cell communication	3.30E-08	429
22	GO:0009966	Regulation of signal transduction	4.63E-08	388

23	GO:0048856	Anatomical structure development	1.01E-07	566
24	GO:0023051	Regulation of signaling	1.29E-07	424
25	GO:0051239	Regulation of multicellular organismal process	1.38E-07	350
26	GO:0080090	Regulation of primary metabolic process	2.75E-07	675
27	GO:0001944	Vasculature development	4.47E-07	92
28	GO:0048514	Blood vessel morphogenesis	1.03E-06	76
29	GO:0001568	Blood vessel development	1.07E-06	88
30	GO:0009893	Positive regulation of metabolic process	1.29E-06	465
31	GO:0060255	Regulation of macromolecule metabolic process	1.65E-06	672
32	GO:0050794	Regulation of cellular process	1.86E-06	1137
33	GO:0009891	Positive regulation of biosynthetic process	1.98E-06	256
34	GO:0006950	Response to stress	1.99E-06	423
35	GO:0010604	Positive regulation of macromolecule metabolic process	2.02E-06	368
36	GO:0010557	Positive regulation of macromolecule biosynthetic process	2.06E-06	237
37	GO:1902531	Regulation of intracellular signal transduction	2.51E-06	271
38	GO:0031328	Positive regulation of cellular biosynthetic process	2.68E-06	252
39	GO:0048731	System development	2.85E-06	483
40	GO:0001817	Regulation of cytokine production	3.18E-06	101
41	GO:0031325	Positive regulation of cellular metabolic process	3.85E-06	381
42	GO:0030968	Endoplasmic reticulum unfolded protein response	4.14E-06	34
43	GO:0050789	Regulation of biological process	5.32E-06	1176
44	GO:0035967	Cellular response to topologically incorrect protein	5.53E-06	36
45	GO:0080135	Regulation of cellular response to stress	6.38E-06	111
46	GO:0034976	Response to endoplasmic reticulum stress	6.64E-06	47
47	GO:0042221	Response to chemical	8.43E-06	459

48	GO:0034620	Cellular response to unfolded protein	9.12E-06	34
49	GO:0042127	Regulation of cell proliferation	1.15E-05	216
50	GO:0033993	Response to lipid	1.26E-05	135

Supplemental Table 7. Enriched signaling pathways in $CdCl_2$-treated H9-CMs

Number	Signaling Pathways	P Value	Q Value
1	Protein processing in endoplasmic reticulum	1.22E-07	2.92E-05
2	Rheumatoid arthritis	6.22E-07	7.43E-05
3	Epithelial cell signaling in Helicobacter pylori infection	3.12E-06	2.49E-04
4	MAPK signaling pathway	3.28E-05	1.78E-03
5	Collecting duct acid secretion	3.73E-05	1.78E-03
6	Legionellosis	0.000139904	5.57E-03
7	NF-kappa B signaling pathway	0.000247382	7.57E-03
8	Nicotinate and nicotinamide metabolism	0.000253553	7.57E-03
9	Vibrio cholerae infection	0.000381183	1.01E-02
10	Osteoclast differentiation	0.000440402	1.05E-02
11	Phagosome	0.001085006	2.36E-02
12	Cytokine-cytokine receptor interaction	0.002083389	4.15E-02
13	African trypanosomiasis	0.002899619	5.33E-02
14	Gap junction	0.003419196	5.57E-02
15	Synaptic vesicle cycle	0.003498379	5.57E-02
16	Mineral absorption	0.004448493	6.25E-02
17	NOD-like receptor signaling pathway	0.004448493	6.25E-02
18	Bladder cancer	0.007702598	1.02E-01
19	Prion diseases	0.009150775	1.15E-01
20	Mucin type O-Glycan biosynthesis	0.0100424	1.20E-01
21	Transcriptional misregulation in cancer	0.0117603	1.34E-01
22	Cholinergic synapse	0.01243281	1.35E-01

23	Pathogenic Escherichia coli infection	0.01303325	1.35E-01
24	Lysosome	0.01586353	1.58E-01
25	MAPK signaling pathway - fly	0.01759887	1.68E-01
26	Hematopoietic cell lineage	0.02000961	1.84E-01
27	Pathways in cancer	0.02188694	1.94E-01
28	Focal adhesion	0.02315032	1.98E-01
29	Salmonella infection	0.02539881	2.09E-01
30	Endocytosis	0.02746478	2.12E-01
31	Wnt signaling pathway	0.0275119	2.12E-01
32	Non-small cell lung cancer	0.02925291	2.12E-01
33	ABC transporters	0.02931757	2.12E-01
34	Prostate cancer	0.03360919	2.29E-01
35	ErbB signaling pathway	0.0342175	2.29E-01
36	Melanoma	0.03446753	2.29E-01
37	Alcoholism	0.03662402	2.34E-01
38	HTLV-I infection	0.03715775	2.34E-01
39	Regulation of actin cytoskeleton	0.04439833	2.66E-01
40	Influenza A	0.04451081	2.66E-01
41	Systemic lupus erythematosus	0.05147255	3.00E-01
42	Amoebiasis	0.05320172	3.03E-01
43	Glioma	0.06327894	3.52E-01
44	Amphetamine addiction	0.06513027	3.54E-01
45	Jak-STAT signaling pathway	0.06691186	3.55E-01
46	Aldosterone-regulated sodium reabsorption	0.07374997	3.77E-01
47	Endometrial cancer	0.07420436	3.77E-01

48	Metabolism of xenobiotics by cytochrome P450	0.07752068	3.86E-01
49	Apoptosis	0.08384827	4.06E-01
50	Renin-angiotensin system	0.08483778	4.06E-01
